# Messenger RNA delivery of a dual CD25/CD122 affinity-tuned IL-2 variant prolongs exposure and potentiates antitumor T cell immunity

**DOI:** 10.1126/sciadv.aeh9215

**Published:** 2026-09-04

**Authors:** Mathias Vormehr, Lena M. Kranz, Alexander Muik, Sina Fellermeier-Kopf, Jan D. Beck, David Eisel, Nadja Salomon, Bonny Gaby Lui, Jan Diekmann, Özlem Türeci, Ugur Sahin

**Affiliations:** ^1^ BioNTech SE, Mainz, Germany.; ^2^ TRON gGmbH – Translational Oncology at the University Medical Center of the Johannes Gutenberg University, Mainz, Germany.; ^3^ HI-TRON – Helmholtz Institute for Translational Oncology Mainz (HI-TRON Mainz) of the DKFZ, Mainz, Germany.

## Abstract

The therapeutic potential of interleukin-2 (IL-2) in cancer treatment is limited by toxicity challenges, partly due to an unfavorable pharmacokinetic profile and unintended activation of regulatory T (T_reg_) cells alongside the desired activation of CD8^+^ effector T cells. To selectively stimulate CD8^+^ T cells over T_reg_ cells, we engineered an IL-2 variant (IL-2var) with a dual-tuned affinity profile that features reduced binding to IL-2Rα (CD25) and enhanced binding to IL-2Rβ (CD122). To optimize its pharmacokinetics and facilitate tumor enrichment, the variant is fused to albumin and delivered as an mRNA encapsulated in a lipid nanoparticle (Alb–IL-2var RNA-LNP), enabling sustained systemic exposure from hepatic production upon intravenous administration. We show that Alb–IL-2var has a favorable pharmacokinetic profile and is tolerated at biologically active doses in immunocompetent mice and cynomolgus monkeys. Selective enhancement of CD8^+^ T cell responses over T_reg_ cells is demonstrated in vitro in human peripheral blood mononuclear cells and in vivo in mice and cynomolgus monkeys. When combined with an mRNA cancer vaccine in syngeneic subcutaneous mouse tumor models, Alb–IL-2var RNA-LNP stimulates the expansion of tumor-infiltrating and circulating tumor antigen–specific CD8^+^ T cells, but not T_reg_ cells. In advanced and cold syngeneic tumor models, it enhances the efficacy of radiotherapy, checkpoint inhibitors, and cancer vaccines. Combination with checkpoint inhibitors and vaccine induces profound proinflammatory conversion of cold tumors. These preclinical results validate a rational design approach to overcome the limitations of IL-2 therapy and support the clinical evaluation of Alb–IL-2var RNA-LNP for solid cancers.

## INTRODUCTION

Interleukin-2 (IL-2) is a key regulator of T cell immunity and antitumor responses (*1*). IL-2 has long been pursued as a cancer therapy, as monotherapy and in combination with CD8^+^ T cell–based approaches including checkpoint blockade, cancer vaccines, and adoptive T cell therapy (*2*). High-dose IL-2 is approved for renal cell carcinoma and metastatic melanoma. However, its use is hindered by two limitations: concurrent expansion of regulatory T (T_reg_) cells alongside cytotoxic CD8^+^ T cells (*3*), and a short serum half-life (*4*, *5*) that requires frequent and high-dose administration, which can lead to severe toxicities such as capillary leak syndrome (*3*, *6*–*9*).

IL-2’s concurrent expansion of T_reg_ and CD8^+^ T cells reflects differential IL-2 receptor (IL-2R) expression across T cell subsets. The high-affinity IL-2R comprises α, β, and γ chains (CD25, CD122, and CD132), whereas the intermediate-affinity receptor contains only β and γ chains (*10*). Naïve CD8^+^ T cells express intermediate-affinity IL-2Rβγ and only transiently up-regulate high-affinity IL-2Rαβγ when activated (*10*, *11*). In contrast, T_reg_ cells constitutively express high levels of IL-2Rαβγ, efficiently capturing IL-2 at low concentrations (*12*) and outcompeting CD8^+^ T cells, thereby promoting T_reg_ cell proliferation and suppressive function. To overcome this, multiple IL-2 variants have been developed to enhance binding to IL-2Rβ (so-called “superkines”) (*13*), reduce binding to the IL-2Rα (so-called “non-alpha” variants) (*14*–*17*), or combine both increased IL-2Rβ binding with completely abrogated IL-2Rα binding (*18*). Reduced IL-2Rβ binding (*19*) and increased IL-2Rα binding have also been explored (*20*) but resulted in either nonspecific expansion of effector T cells or T_reg_ cell expansion.

Herein, we report the rational design of IL-2var, a novel IL-2 variant engineered to selectively amplify antigen-specific CD8^+^ T cells while limiting T_reg_ cell expansion. Its design was guided by the different expression profiles of IL-2Rs in T cell subsets. Antigen-activated effector CD8^+^ T cells transiently up-regulate IL-2Rα and maintain elevated IL-2Rβ expression (*21*), yielding a CD25^high^CD122^high^ phenotype that distinguishes them from T_reg_ cells (CD25^very high^CD122^int^), naïve CD8^+^ T cells (CD25^neg^CD122^int^), and resting memory CD8^+^ T cells (CD25^neg^CD122^high^) (*22*). We therefore designed IL-2var to have increased IL-2Rβ binding and attenuated IL-2Rα binding, in contrast to “non-α” IL-2 variants that abolish IL-2Rα binding. This dual-tuning approach is intended to favor antigen-activated CD8^+^ T cells over other lymphocytes. We therefore anticipate that IL-2var may be especially effective when combined with immunotherapies that induce or enhance antitumor T cell responses, such as mRNA cancer vaccines or checkpoint inhibitors.

To address the short serum half-life of IL-2, we built upon our previous design of mRNA-encoded albumin–IL-2 fusion proteins (*23*, *24*). Formulation in lipid nanoparticles (LNPs) enables intravenous administration, liver uptake, and sustained expression, prolonging systemic exposure while avoiding high peak concentrations associated with toxicity (*25*). Albumin fusion further extends protein serum half-life and favors enrichment in tumor tissue through enhanced permeability and retention (*23*, *26*). To facilitate clinical translation, we engineered mRNA encoding albumin-fused IL-2var (Alb–IL-2var) with optimized untranslated elements and encapsulated it in a clinical-grade LNP formulation.

We present a preclinical characterization of Alb–IL-2var RNA-LNP. In animal models, it selectively expanded tumor-specific CD8^+^ T cells and enhanced T cell–dependent therapies including cancer vaccines and checkpoint blockade. These findings support our dual-tuning design concept and identify RNA-LNP–encoded albumin-cytokine fusion proteins (“RiboCytokines”) as a potential alternative to recombinant cytokine therapy. Based on these data, a phase 1 trial of Alb–IL-2var RNA-LNP (NCT04455620) was initiated.

## RESULTS

### IL-2 variants with partially retained IL-2Rα binding and increased IL-2Rβ binding selectively enhance stimulation of CD8^+^ effector T cells in culture and in mice

**Fig. 1.**
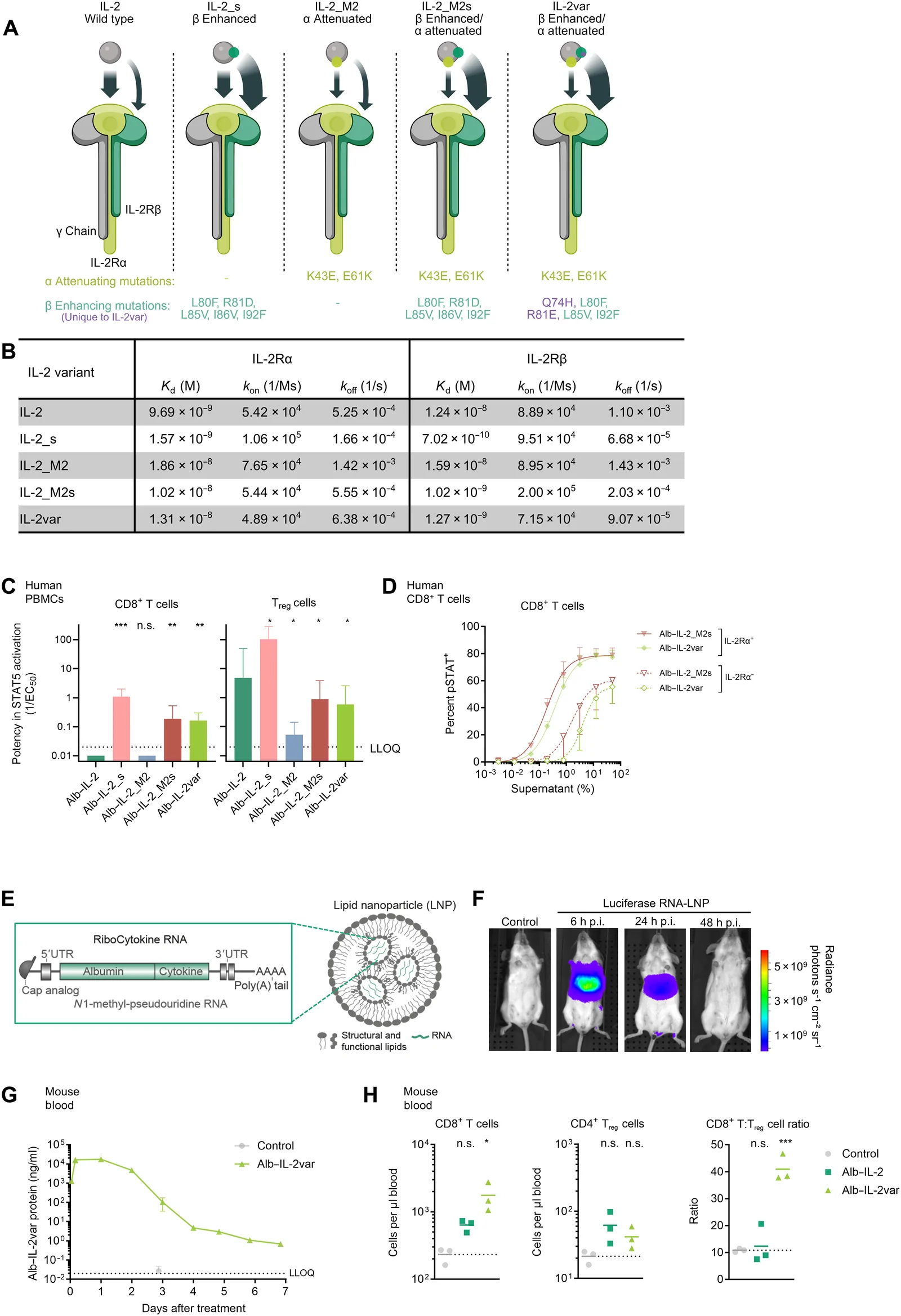
IL-2 variants with partially retained IL-2Rα binding and increased IL-2Rβ binding selectively enhance stimulation of CD8^+^ T cells in cell culture and in mice. (**A**) IL-2 variants evaluated in this study. (**B**) Affinity measurements of IL-2 variants to IL-2Rα and IL-2Rβ as determined by biolayer interferometry. *K*_d_, dissociation constant. (**C**) Signal transducer and activator of transcription 5 (STAT5) phosphorylation in T cell subsets within human peripheral blood mononuclear cells (PBMCs; *n* = 5 donors) was analyzed following stimulation with serial dilutions of human embryonic kidney (HEK) 293T supernatants containing Alb–IL-2 variants, as determined by flow cytometry. The potency of Alb–IL-2 variants is expressed as reciprocal median effective concentration (EC_50_). For potencies below the lower limit of quantification (LLOQ), LLOQ/2 values were used for EC_50_. (**D**) STAT5 phosphorylation in IL-2Rα RNA-electroporated or nonelectroporated CD8^+^ T cells isolated from PBMCs (*n* = 2 donors) upon incubation with HEK293T supernatants containing Alb–IL-2 variants as determined by flow cytometry. (**E**) RiboCytokine platform technology. Albumin-cytokine fusion protein sequences are encoded by modified single-stranded RNA and formulated into LNPs. (**F**) Bioluminescence in vivo imaging of BALB/c mice (*n* = 3) injected intravenously with 1 μg of firefly luciferase RNA-LNP. Alb RNA-LNP served as control. h, hours. (**G**) Serum levels of translated Alb-IL–2var protein in BALB/c mice (*n* = 3) injected intravenously with 10 μg of Alb–IL-2var RNA-LNP (Alb–IL-2var). Alb RNA-LNP served as the control. (**H**) Flow cytometry analysis of CD8^+^ and CD4^+^ T cells, T_reg_ cells and CD8^+^ T–to–T_reg_ cell ratio in blood of C57BL/6 mice (*n* = 3) 7 days after dosing with 10 μg of Alb–IL-2 RNA-LNP (Alb–IL-2) or Alb–IL-2var RNA-LNP (Alb–IL-2var). Alb RNA-LNP served as the control. Horizontal dotted lines represent the means of the control group (H). One-way analysis of variance (ANOVA) with Dunnett’s post hoc test (C and H). Not significant (n.s.), *P* > 0.05; *P ≤ 0.05; **P ≤ 0.01; ***P ≤ 0.001.

To identify an IL-2 variant with enhanced affinity for IL-2Rβ and reduced affinity for IL-2Rα, which would selectively stimulate CD8^+^ T cells over T_reg_ cells, we introduced distinct sets of mutations into human IL-2 and assessed their effects on CD8^+^ T cell and T_reg_ cell activation. We constructed four IL-2 variants: (i) IL-2_s, which incorporated five previously described β-enhancing mutations in the cytokine’s core (*13*); (ii) IL-2_M2, in which two of five ion-pair interactions at the IL-2/IL-2Rα interface (*10*) were disrupted to reduce IL-2Rα binding; (iii) IL-2_M2s, which combined both mutation sets; and (iv) IL-2var, a refined version of IL-2_M2s in which two of the β-enhancing mutations were replaced with alternative substitutions to further optimize receptor binding characteristics (Fig. 1A) (*27*). Affinity measurements confirmed that, whereas IL-2_s exhibited increased affinity for IL-2Rβ, IL-2_M2 showed markedly reduced, but not fully abrogated, binding to IL-2Rα. Both IL-2_M2s and IL-2var demonstrated the intended dual profile of enhanced IL-2Rβ binding and attenuated IL-2Rα interaction (Fig. 1B).

To evaluate the functional consequences of these modifications, human embryonic kidney (HEK) 293T cells were transfected with RNA encoding albumin (Alb) fusions of IL-2 and the four IL-2 variants. Supernatants containing the secreted proteins were collected and tested for their potency to activate primary human T cell subsets, as determined by signal transducer and activator of transcription 5 (STAT5) phosphorylation displayed as reciprocal median effective concentration (EC_50_) values. As expected, Alb–IL-2 induced markedly higher STAT5 phosphorylation in T_reg_ cells compared with that in CD8^+^ T cells, with activation in the latter remaining below the lower limit of quantification. Relative to Alb–IL-2, Alb–IL-2_s induced significantly higher STAT5 phosphorylation in CD8^+^ T cells and T_reg_ cells, whereas Alb–IL-2_M2 had no effect on CD8^+^ T cell activation and induced significantly lower STAT5 phosphorylation in T_reg_ cells. In contrast, Alb–IL-2var and Alb–IL-2_M2s demonstrated more than 10-fold greater potency in activating CD8^+^ T cells and more than 10-fold lower stimulation of T_reg_ cells relative to Alb–IL-2, with all differences reaching statistical significance (Fig. 1C). Notably, Alb–IL-2var or Alb–IL-2_M2s maintained some affinity for IL-2Rα, as demonstrated by their 11.2- and 8.8-fold higher activation of IL-2Rα–RNA–electroporated CD8^+^ T cells compared with that of nonelectroporated CD8^+^ T cells (Fig. 1D and fig. S1).

With Alb–IL-2var and Alb–IL-2_M2s exhibiting comparable receptor binding affinity and functional profiles, we selected Alb–IL-2var for further investigation. To evaluate the pharmacology of Alb–IL-2var in vivo, we used our RNA-LNP platform (Fig. 1E), which enables selective and high protein expression by the liver within 6 hours of injection and sustained detectability of robust protein signals after 24 hours, as shown by luciferase reporter RNA translation (Fig. 1F and fig. S2). Following intravenous administration of 10 μg of Alb–IL-2var RNA-LNP (∼500 μg/kg) in BALB/c mice, Alb–IL-2var protein was detected in serum within 1 hour, with a mean half-life of 23.3 hours and peak concentration (*C*_max_) of 17.6 μg/ml at 24 hours (Fig. 1G).

To assess the immunological effects of Alb–IL-2var, C57BL/6 mice were dosed with Alb–IL-2var RNA-LNP or Alb–IL-2 RNA-LNP. Analysis of peripheral blood T cells after a single treatment revealed that Alb–IL-2var RNA-LNP significantly expanded CD8^+^ T cells compared with those of control mice, with a greater effect than Alb–IL-2 RNA-LNP. In contrast, Alb–IL-2 RNA-LNP induced more expansion of T_reg_ cells than Alb–IL-2var RNA-LNP. Consequently, the CD8^+^ T–to–T_reg_ cell ratio was significantly higher in mice treated with Alb–IL-2var RNA-LNP than in those treated with Alb–IL-2 RNA-LNP (Fig. 1H).

### Alb–IL-2var is superior to Alb–IL-2 in expanding vaccine-induced CD8^+^ T cells and mediating complete tumor regression in mice

A key therapeutic application of IL-2 is the expansion of antigen-specific T cells induced by cancer vaccines (*28*). To evaluate the therapeutic potential of Alb–IL-2var RNA-LNP in combination with a cancer vaccine, we used the lipoplex-formulated RNA (RNA-LPX) vaccine platform (*29*), which is now under investigation in multiple clinical trials (NCT06534983, NCT05968326, NCT04486378, and NCT05557591). RNA-LPX is selectively taken up by dendritic cells resident in lymphoid compartments, where the RNA is translated into encoded tumor antigens, leading to the induction of tumor-specific T cells and durable antitumor effects in mice (*29*–*32*) and humans (*33*–*36*).

We have previously shown in CT26 and B16F10 mouse tumor models that RNA-encoded Alb–IL-2 boosts the expansion, effector cytokine production, cytotoxicity, and antitumor activity of RNA-LPX vaccine–induced CD8^+^ T cells (*23*). Building on these findings, we hypothesized that IL-2var, by dampening peripheral T_reg_ cell responses, might further amplify these vaccine-driven effects.

**Fig. 2.**
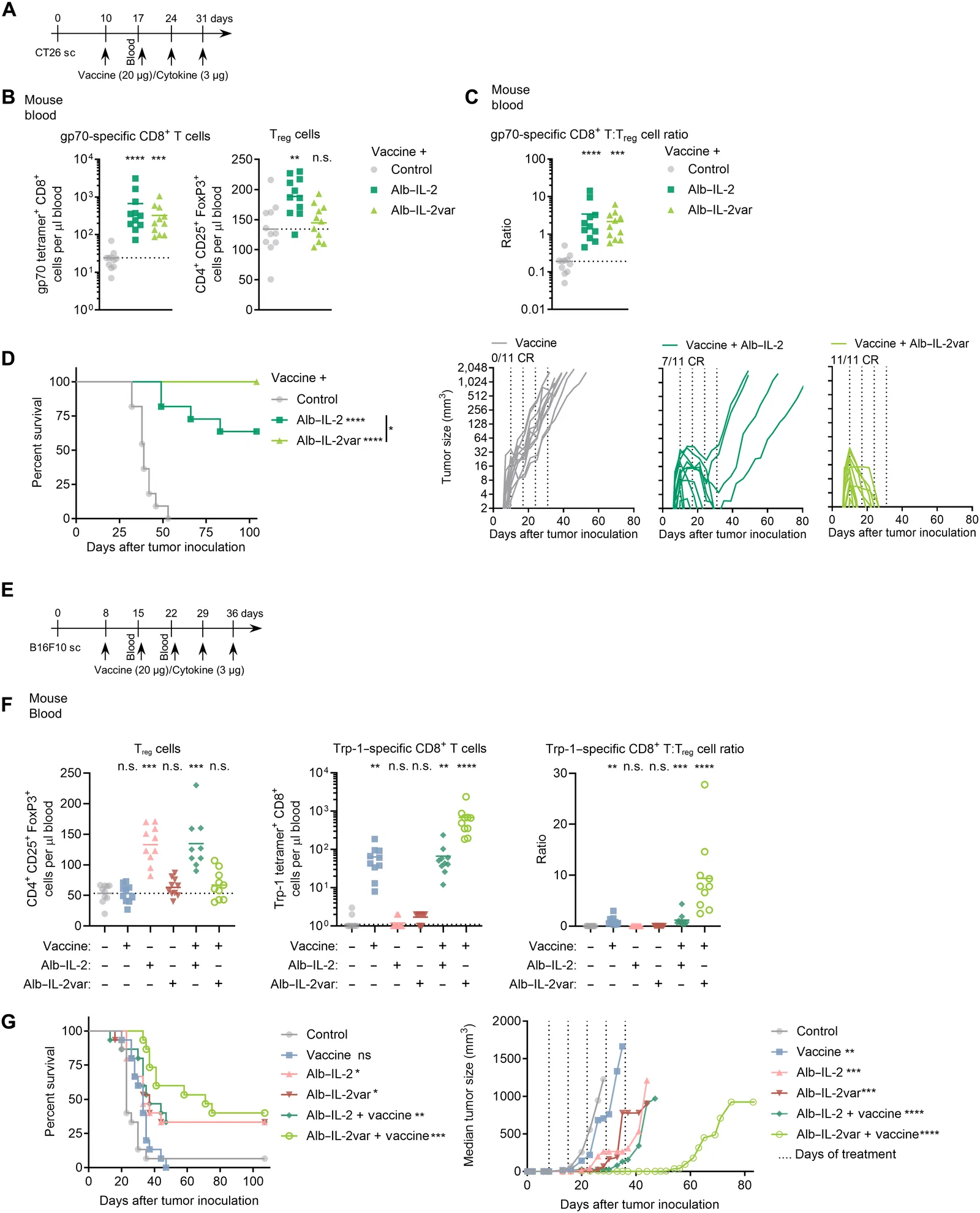
Alb–IL-2var is superior to Alb–IL-2 in expanding vaccine-induced CD8^+^ T cells and mediating complete tumor regression in mice. (**A** to **D**) CT26 tumor-bearing BALB/c mice (*n* = 11) were treated with gp70 RNA-LPX vaccine (vaccine), Alb–IL-2 RNA-LNP (Alb–IL-2), or Alb–IL-2var RNA-LNP (Alb–IL-2var), starting on day 10 post–tumor inoculation (average tumor volume, 16 mm^3^). Alb RNA-LNP served as the control. (A) Experimental design. (B) gp70-specific CD8^+^ T and T_reg_ cells, and (C) gp70-specific CD8^+^ T–to–T_reg_ cell ratio in blood 7 days after the first treatment, as determined by flow cytometry. (D) Survival and individual tumor growth curves. Horizontal dotted lines represent the means of the control group (B and C), and vertical dotted lines represent treatments (D). (**E** to **G**) B16F10 tumor-bearing C57BL/6 mice (*n* = 15) were treated with Trp-1 RNA-LPX vaccine (vaccine), Alb–IL-2 RNA-LNP (Alb–IL-2), and/or Alb–IL-2var RNA-LNP (Alb–IL-2var), starting on day 8 post–tumor inoculation (average tumor volume, 3 mm^3^). Irrelevant RNA-LPX and hAlb RNA-LNP served as a control. (E) Experimental design. (F) Trp-1–specific CD8^+^ T cells 7 days after the second treatment, T_reg_ cells 7 days after the first treatment, and CD8^+^ T–to–T_reg_ cell ratio 7 days after the second treatment, in blood, as determined by flow cytometry. (G) Survival and individual tumor growth curves. Horizontal dotted lines represent the means of the control group (F), and vertical dotted lines represent treatments (G). Statistical tests: Kruskal-Wallis test with Dunn’s multiple comparison test (B, C, and F), log-rank test (D, left; and G, left), and two-way ANOVA with Dunnett’s multiple comparison test (G, right). Not significant (n.s.), *P* > 0.05; *P ≤ 0.05; **P ≤ 0.01; ***P ≤ 0.001; ****P ≤ 0.0001. CR, complete regression; sc, subcutaneous; Trp-1, tyrosinase-related protein 1.

The CT26 colon carcinoma model is highly immunogenic and expresses the viral antigen glycoprotein 70 (gp70) derived from the endogenous Moloney murine leukemia virus, which is a well-characterized target for CD8^+^ T cells. CT26 tumor-bearing mice were treated weekly with Alb–IL-2 RNA-LNP or Alb–IL-2var RNA-LNP, in combination with a gp70-epitope–encoding RNA-LPX vaccine (Fig. 2A). Both Alb–IL-2 RNA-LNP and Alb–IL-2var RNA-LNP induced significant expansion of preexisting (*24*) gp70-specific CD8^+^ T cells after a single treatment, with Alb–IL-2 showing slightly higher activity (Fig. 2B). However, Alb–IL-2 also significantly expanded T_reg_ cells, whereas Alb–IL-2var did not. Animals receiving the vaccine in combination with Alb–IL-2 RNA-LNP or Alb–IL-2var RNA-LNP had significantly elevated antigen-specific CD8^+^ T–to–T_reg_ cell ratios compared with the vaccine alone (Fig. 2C).

Combining Alb–IL-2 RNA-LNP or Alb–IL-2var RNA-LNP with the vaccine resulted in tumor rejections, which were not observed with the vaccine alone, and provided significant survival benefit compared with that in control animals (Fig. 2D). Most animals that received Alb–IL-2 RNA-LNP and the vaccine experienced sustained tumor rejections (7 of 11 mice; 63.5%). By contrast, all mice treated with a combination of Alb–IL-2var RNA-LNP and the vaccine rejected their tumors and remained tumor-free throughout the posttreatment observation period, resulting in significantly improved survival compared with animals treated with Alb–IL-2 RNA-LNP and the vaccine. The observed antitumor activity of Alb–IL-2var RNA-LNP in combination with the vaccine required both components, as demonstrated in a complementary experiment using monotherapy controls in the same tumor model (fig. S3A). Phenotypic profiling of vaccine-induced CD8^+^ T cells in the blood revealed that Alb–IL-2var RNA-LNP did not alter the proportions of KLRG1^−^CD127^+^ memory-precursor or KLRG1^+^CD127^−^ short-lived effector cells, which were primarily shaped by the RNA-LPX vaccine, but significantly increased the KLRG1^+^CD127^+^ subset, reported to down-regulate KLRG1 and contribute to the memory pool (fig. S3B) (*37*). In line with the substantial antitumor activity of the combination treatment, Alb–IL-2var RNA-LNP significantly reduced the fraction of PD-1^+^ CD8^+^ T cells coexpressing the exhaustion-associated transcription factor TOX among RNA-LPX vaccine–induced CD8^+^ T cells in blood and spleen (fig. S3C) (*38*, *39*).

We next tested Alb–IL-2 RNA-LNP and Alb–IL-2var RNA-LNP in the poorly immunogenic B16F10 melanoma model that forms aggressive, difficult-to-treat tumors (*40*). Alb–IL-2 RNA-LNP and Alb–IL-2var RNA-LNP were administered weekly either as monotherapy or in combination with a vaccine that encoded an epitope of the tumor-associated antigen tyrosinase-related protein (Trp) (Fig. 2E) (*41*). A single treatment with Alb–IL-2 RNA-LNP resulted in significant T_reg_ cell expansion in the blood, whereas Alb–IL-2var RNA-LNP had no significant effect on T_reg_ cell numbers, regardless of vaccination. Due to the absence of preexisting Trp-1–specific T cells, these cells became detectable in the blood only after two immunizations. Notably, Alb–IL-2var RNA-LNP amplified vaccine-induced Trp-1–specific T cell expansion by more than 10-fold, whereas Alb–IL-2 RNA-LNP had little effect. In Alb–IL-2var RNA-LNP–treated mice, this resulted in a nearly 10-fold higher antigen-specific CD8^+^ T–to–T_reg_ cell ratio than the vaccine administered alone or in combination with Alb–IL-2 RNA-LNP (Fig. 2F). The survival of B16F10 tumor-bearing mice was significantly improved by treatment with either Alb–IL-2var RNA-LNP (median survival of 37 days; complete tumor regression in 33%) or Alb–IL-2 RNA-LNP (33 days; 33%) compared with the control group (23 days; 7%), whereas the vaccine alone yielded a nonsignificant improvement (33 days; 0%) (Fig. 2G). Relative to their respective monotherapies, combining the vaccine with Alb–IL-2var RNA-LNP (median survival of 71 days; 40%) more significantly extended survival, whereas combining it with Alb–IL-2 RNA-LNP (37 days; 33%) provided little additional benefit. Likewise, mice treated with the combination of Alb–IL-2var RNA-LNP and the vaccine exhibited the slowest tumor growth of all groups. Although treatment with single-agent Alb–IL-2 RNA-LNP delayed tumor growth, combining Alb–IL-2 RNA-LNP with the vaccine failed to confer further benefit.

In summary, these findings from two immunologically distinct tumor models substantiate the capacity of Alb–IL-2var RNA-LNP to stimulate the expansion of tumor antigen–specific CD8^+^ T cells and highlight its robust therapeutic efficacy, both as a standalone treatment or in combination with cancer vaccines.

### Alb–IL-2var enhances antitumor efficacy of anti–PD-L1 alone and in combination with LRT

Another application of IL-2 therapy is the combination with PD-1/PD-L1 immune checkpoint inhibition (CPI). A previous study demonstrated that a recombinant IL-2 variant harboring a set of mutations distinct from IL-2var that completely abolishes IL-2Rα binding preferentially promotes bystander CD8^+^ T cell proliferation but fails to activate antigen-specific CD8^+^ T cells and, consequently, does not synergize with PD-1/PD-L1 CPI (*42*). We hypothesized that Alb–IL-2var, due to its residual IL-2Rα affinity, may retain the capacity to stimulate tumor-reactive CD8^+^ T cells that transiently express IL-2Rα upon antigen encounter (*43*).

To assess the contribution of Alb–IL-2var to CPI treatment, we explored three therapeutic concepts: (i) combination with an anti–PD-L1 antibody to enhance reinvigorated, antigen-specific T cell responses; (ii) combination with anti–PD-L1 following local radiotherapy (LRT) to support immunity against antigens released through radiation-induced cell death (*32*); and (iii) triple combination with vaccine and anti–PD-L1 to boost vaccine-induced, checkpoint-blockade-supported immunity.

For the first two study designs, we used the immunogenic MC38 tumor model. MC38 is a mouse colon adenocarcinoma with a high mutational burden (*44*), rendering it responsive to PD-1/PD-L1 CPI (*24*). Multiple neoantigens have been described in this model, including immunogenic nonsynonymous single-nucleotide variants in the genes *Adpgk* (*45*), *Rpl18* (*24*, *46*), and *N4bp2l2* (*47*). Among these, mutated *Rpl18* is characterized by high gene expression levels and elicits the strongest spontaneous immune responses, whereas mutated Adpgk-specific T cell responses were reported to dominate vaccine-induced immunity (*44*–*46*).

**Fig. 3.**
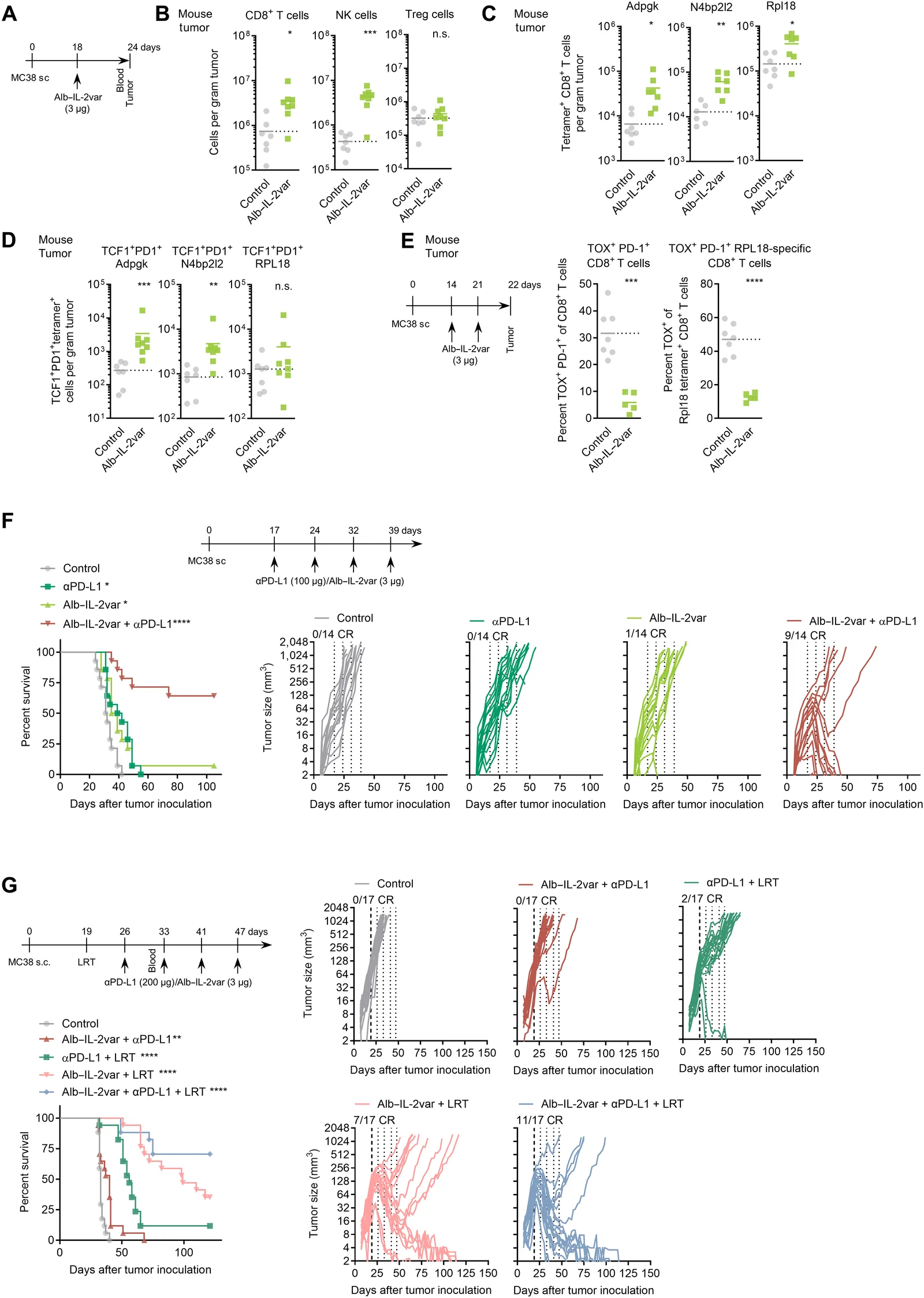
Alb–IL-2var enhances antitumor efficacy of anti–PD-L1 alone and in combination with LRT. (**A** to **D**) MC38 tumor-bearing C57BL/6 mice (*n* = 7 to 8) were treated with Alb–IL-2var RNA-LNP (Alb–IL-2var) starting on day 18 post–tumor inoculation (average tumor volume, 165 mm^3^). Alb RNA-LNP served as a control. (A) Experimental design. (B) Flow cytometry analysis of tumor-infiltrating total CD8^+^ T cells, natural killer (NK) cells, and T_reg_ cells. (C) Flow cytometry analysis of neoantigen-specific CD8^+^ T cells. (D) TCF1^+^PD-1^+^ neoantigen-specific CD8^+^ T cells, as determined by flow cytometry. (**E**) MC38 tumor-bearing C57BL/6 mice (*n* = 5 to 7) were treated with Alb–IL-2var RNA-LNP (Alb–IL-2var) on days 14 and 21 post–tumor inoculation (average tumor volume, 86 mm^3^). Alb RNA-LNP served as a control. TOX^+^PD-1^+^ cells among tumor-infiltrating CD8^+^ T cells (left) and Rpl18-specific CD8^+^ T cells (right), as determined by flow cytometry. (B to E) Horizontal dotted lines represent the means of the control group. (**F**) Survival and individual tumor growth curves of MC38 tumor-bearing C57BL/6 mice (*n* = 14) treated with Alb–IL-2var RNA-LNP (Alb–IL-2var), and/or anti–PD-L1 antibody (αPD-L1) starting on day 17 post–tumor inoculation (average tumor volume, 73 mm^3^). Alb RNA-LNP and isotype antibody served as controls. Vertical dotted lines represent treatments. (**G**) Survival and individual tumor growth curves of MC38 tumor-bearing C57BL/6 mice (*n* = 17) treated with LRT, Alb–IL-2var RNA-LNP (Alb–IL-2var), and αPD-L1. LRT began on day 19 post–tumor inoculation (average tumor volume, 90 mm^3^), whereas Alb–IL-2var RNA-LNP and/or αPD-L1 were administered on day 26, when tumors exceeded 200 mm^3^. Vertical dashed line represents LRT, and vertical dotted lines represent αPD-L1/Alb–IL-2var RNA-LNP treatments. Alb RNA-LNP and isotype antibody served as controls. Student’s unpaired *t* test (A to E) and log-rank test (F and G, left). Single outliers as identified by Grubb’s test were removed (B). Not significant, *P* > 0.05; *P ≤ 0.05; **P ≤ 0.01; ***P ≤ 0.001; ****P ≤ 0.0001.

Typically, MC38 tumors have a notable preexisting immune infiltrate (*24*). Following a single dose of Alb–IL-2var RNA-LNP (Fig. 3A), we observed significant increases in intratumoral CD8^+^ T cells and natural killer (NK) cells, whereas T_reg_ cell numbers remained unchanged (Fig. 3B). Within the expanded CD8^+^ T cell compartment, neoantigen-specific T cells (typically associated with tumor rejection in this model) were significantly enriched (Fig. 3C). Treatment-induced changes in blood closely mirrored those in the tumor across CD8^+^ T cells, NK cells, T_reg_ cells, and tumor antigen–specific CD8^+^ T cells (fig. S4A). Alb–IL-2var RNA-LNP also increased or maintained the number of intratumoral TCF1^+^PD-1^+^ and reduced the proportion of PD-1^+^TOX^+^ antigen-specific cells (Fig. 3, D and E), consistent with expansion of a stem-like CD8^+^ T cell pool and reduced terminal exhaustion (*38*, *39*, *42*, *48*, *49*).

Next, to test whether these immunologic changes translated into therapeutic benefit, MC38 mice bearing advanced tumors were treated weekly with Alb–IL-2var RNA-LNP and anti–PD-L1 as monotherapies and in combination. The monotherapies modestly improved survival compared with that in the control mice (median survival of 37 days and 41 days, respectively, compared with 32 days in the control group). However, complete tumor regression was only observed in 1 of 14 (7%) mice in the Alb–IL-2var RNA-LNP group and none in the anti–PD-L1 group (Fig. 3F). In contrast, combining Alb–IL-2var RNA-LNP and anti–PD-L1 yielded a markedly more significant improvement in survival, with complete tumor regression observed in 9 of the 14 (64%) mice.

Mice with even more advanced MC38 tumors were treated weekly with Alb–IL-2var RNA-LNP and/or anti–PD-L1, alone or in combination with LRT pretreatment. In this high tumor load setting, combined treatment with Alb–IL-2var RNA-LNP and anti–PD-L1 without LRT provided only minimal survival benefit (median survival of 40 days versus 33 days in controls) and did not result in complete tumor regression (Fig. 3G). Double combination of LRT with either anti–PD-L1 or Alb–IL-2var RNA-LNP more significantly extended survival (median survival of 56 and 99 days, respectively), with complete tumor regression observed in 2 of 17 (12%) and 7 of 17 (41%) mice, respectively. Notably, the triple combination of LRT, Alb–IL-2var RNA-LNP, and anti–PD-L1 yielded the most significant therapeutic benefit, with complete tumor regression observed in 11 of the 17 mice (65%) and median survival therefore not reached. Analysis of peripheral blood on day 33 revealed that expansion of neoantigen-specific CD8^+^ T cells was primarily driven by Alb–IL-2var RNA-LNP (fig. S4B).

### Alb–IL-2var enhances antitumor efficacy of anti–PD-L1 alone and in combination with cancer vaccines

For the third study design assessing the contribution of Alb–IL-2var to the therapeutic effect of vaccine-induced, checkpoint blockade–supported immunity, we used the TC-1 model, a murine lung epithelial cell line transformed with the oncogenic human papillomavirus 16 (HPV16) oncoproteins E6 and E7 (*50*). Although the viral neoantigen E7 contains a strong CD8^+^ T cell epitope, it fails to elicit spontaneous tumor-specific T cell responses in this CPI refractory model (*24*, *31*, *51*, *52*).

**Fig. 4.**
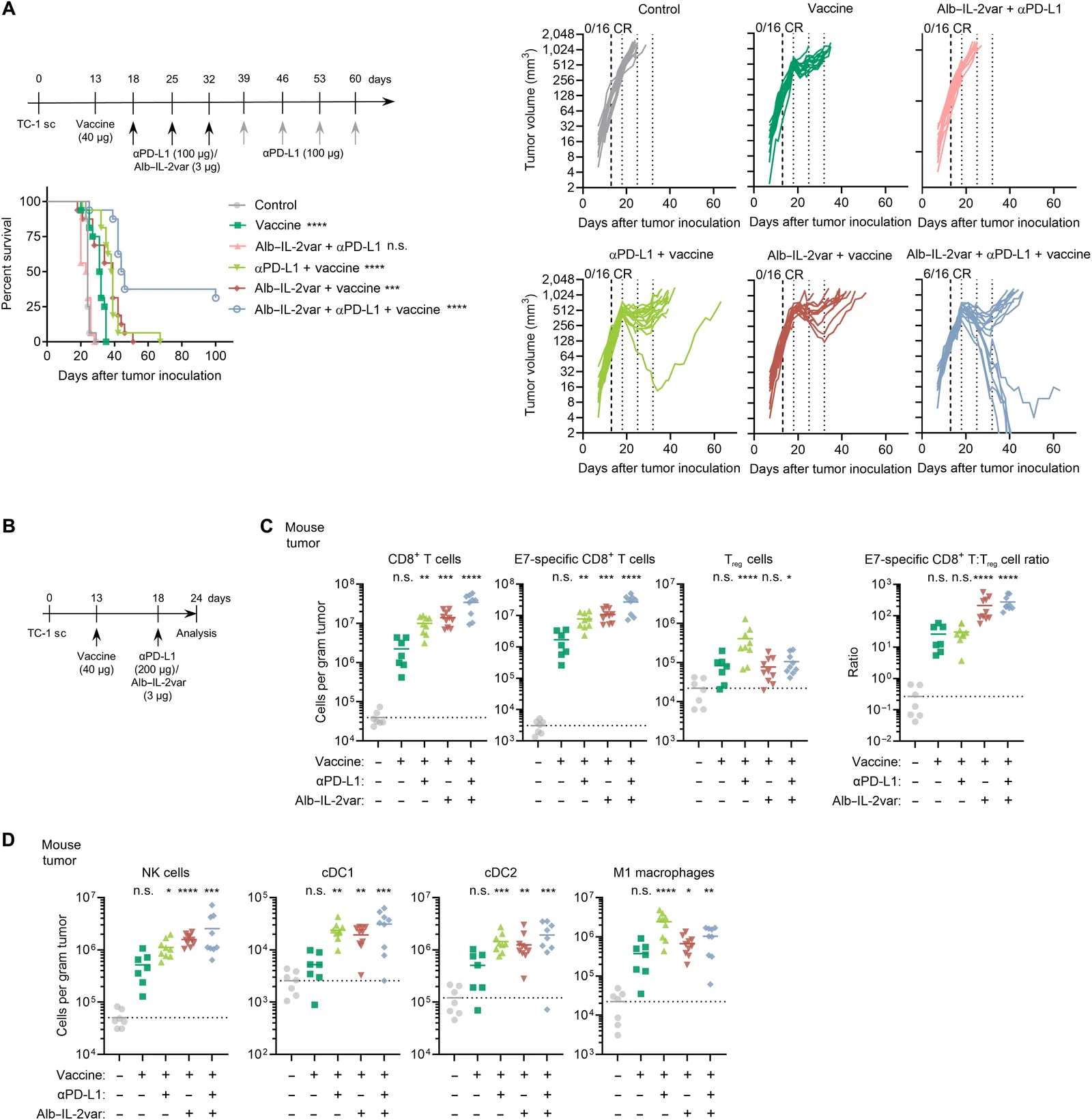
Alb–IL-2var enhances antitumor efficacy of anti–PD-L1 alone and in combination with cancer vaccines. (**A**) Survival and individual tumor growth curves of TC-1 tumor-bearing C57BL/6 mice (*n* = 16) treated with HPV16 E7 RNA-LPX vaccine (vaccine), anti–PD-L1 antibody (αPD-L1), and Alb–IL-2var RNA-LNP (Alb–IL-2var). Vaccination began on day 13 post–tumor inoculation (average tumor volume, 140 mm^3^), whereas Alb–IL-2var RNA-LNP and/or αPD-L1 were administered on day 18, when tumors exceeded 350 mm^3^. Alb RNA-LNP, irrelevant RNA-LPX, and isotype antibody served as controls. Vertical dashed lines represent vaccine treatment, and vertical dotted lines represent αPD-L1 or Alb–IL-2var RNA-LNP treatments. (**B** to **D**) TC-1 tumor-bearing C57BL/6 mice (*n* = 7 to 10) were treated with HPV16 E7 RNA-LPX vaccine (vaccine), anti–PD-L1 antibody (αPD-L), and/or Alb–IL-2var RNA-LNP (Alb–IL-2var) starting on day 13 post–tumor inoculation (average tumor volume, 139 mm^3^). Alb RNA-LNP, irrelevant RNA-LPX, and isotype antibody served as controls. (B) Experimental design. (C) Tumor-infiltrating total- and HPV16 E7-specific CD8^+^ T cells and T_reg_ cells as well as the HPV16 E7-specific CD8^+^ T–to–T_reg_ cell ratio as determined by flow cytometry. (D) Tumor-infiltrating NK cells, cDC1, cDC2, and M1 macrophages as determined by flow cytometry. Horizontal dotted lines represent the means of the control group. Log-rank test (A, left) and Kruskal-Wallis test with Dunn’s multiple comparison test (B to D). Not significant (n.s.), *P* > 0.05; *P ≤ 0.05; **P ≤ 0.01; ***P ≤ 0.001; ****P ≤ 0.0001.

A single HPV16 E7 RNA-LPX vaccine dose administered to TC-1 tumor-bearing mice on day 13 led to transient remission and increased median survival (31.5 days versus 24 days in the control group) (Fig. 4A). When combined with weekly administration of either Alb–IL-2var RNA-LNP or anti–PD-L1 starting on day 18, the therapeutic effect of the vaccine was reinforced, extending median survival to 39 days in both groups. As expected, due to the absence of tumor antigen–specific T cell responses at baseline, the combination of Alb–IL-2var RNA-LNP and anti–PD-L1 without vaccination had no effect on survival or tumor burden (median survival of 24 days). The triple combination of the vaccine, Alb–IL-2var RNA-LNP, and anti–PD-L1 provided the greatest survival benefit (median survival of 45 days), enabling lasting tumor rejection in 37.5% (6 of 16) of the treated mice and delaying tumor growth in the remaining animals.

Among all treatment groups, the triple combination elicited the most robust infiltration of total CD8^+^ T cells and E7-specific CD8^+^ T cells within the tumor. Of note, while T_reg_ cells were significantly expanded with the combination of anti–PD-L1 and vaccine, only a modest increase was observed with the triple combination, which yielded the highest ratio of E7-specific CD8^+^ T to T_reg_ cells (Fig. 4, B and C). In addition, the triple combination led to the strongest expansion of NK cells as well as type 1 and type 2 conventional dendritic cells (cDC1 and cDC2, respectively) and to a significant increase of M1-polarized, proinflammatory macrophages (Fig. 4D).

Collectively, these data demonstrate that Alb–IL-2var enhances the efficacy of CPI therapy across multiple therapeutic contexts by promoting the expansion and function of tumor-specific effector cells while limiting the expansion of T_reg_ cells. Specifically, we identified combinations with LRT being particularly effective for large tumors and combinations with RNA-LPX vaccination contributing to substantial remodeling of immunologically cold tumors.

### Alb–IL-2var demonstrates favorable pharmacokinetic and pharmacodynamic profiles in NHPs

We investigated the immunological effects of Alb–IL-2var in nonhuman primates (NHPs). Naïve female cynomolgus monkeys (*n* = 21 in total) were dosed with Alb–IL-2var RNA-LNP across three pharmacokinetic/pharmacodynamic studies, with doses ranging from 4 to 180 μg/kg. All animals tolerated the treatments well, with no test item–related deaths or sequelae observed. Soft feces were noted for several animals during the study, which was deemed related to treatment administration or stress, and not related to the test item. No major effects were seen on local or systemic tolerance and no effects on body weight, food or water consumption were recorded.

**Fig. 5.**
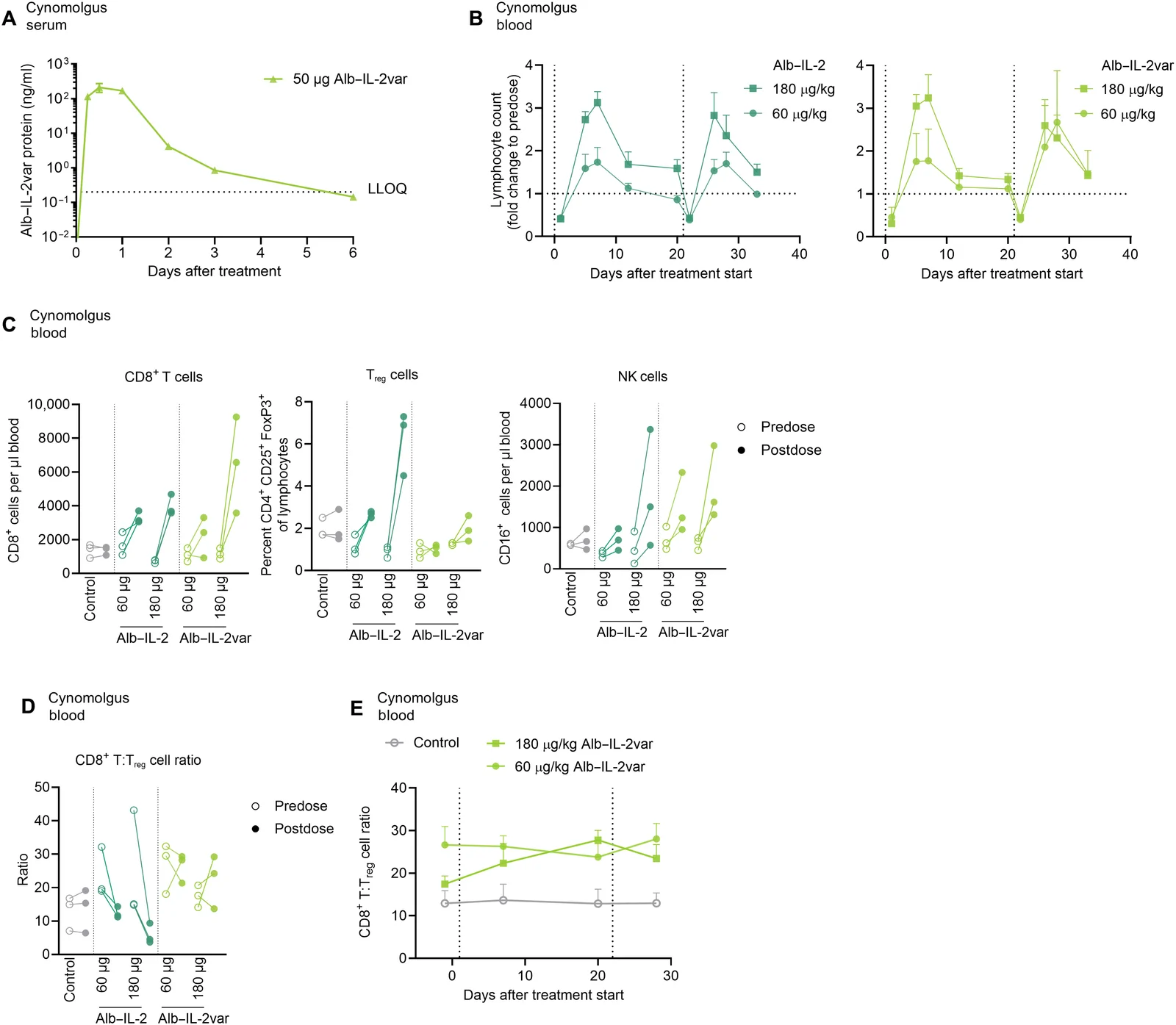
Alb–IL-2var RNA-LNP demonstrates favorable pharmacokinetic and pharmacodynamic profiles in non-human primates. (**A**) Serum levels of translated Alb–IL-2var in female cynomolgus monkeys (*n* = 3) injected intravenously with 50 μg (mean of 19.45 μg/kg) of Alb–IL-2var RNA-LNP (Alb–IL-2var). (**B** to **E**) Lymphocytes as assessed by hematocytometry (means + SEM) (B), CD8^+^ T cells, T_reg_ cells, and NK cells pre- and postdose (7 days after treatment) (C) as well as CD8^+^ T–to–T_reg_ cell ratios pre- and postdose (D) and over time (means + SEM) (E) as determined by flow cytometry in female cynomolgus monkeys (*n* = 3) treated with Alb–IL-2 RNA-LNP (Alb–IL-2) or Alb–IL-2var RNA-LNP (Alb–IL-2var) (60 or 180 μg/kg). Empty LNPs (lipid dose adapted to 120 μg of RNA/kg) served as control. LLOQ, lower limit of quantification.

The pharmacokinetic profile of in vivo–translated Alb–IL-2var in cynomolgus monkeys (Fig. 5A) was similar to that observed in mice (Fig. 1G). A single dose of 50 μg of Alb–IL-2var RNA-LNP (19.45 μg/kg) yielded a serum half-life of 21.3 hours, which is markedly longer than the half-life of rhIL-2 in patients (5 to 7 min) (*53*). A *C*_max_ of 217 ng/ml was reached 16 hours after administration.

In cynomolgus monkeys receiving two doses of either Alb–IL-2var RNA-LNP or Alb–IL-2 RNA-LNP at 60 or 180 μg/kg, 3 weeks apart, peripheral blood lymphocytes declined within 1 day of administration. This was followed by a threefold increase compared with predose values, lasting for at least 7 days, before subsequently declining (Fig. 5B). These fluctuations in lymphocytes after administration of Alb–IL-2var RNA-LNP or Alb–IL-2 RNA-LNP were paralleled by similar dynamics in CD8^+^ T cell and NK cell numbers (Fig. 5C).

Notably, T_reg_ cell frequencies sharply increased by day 8 in Alb–IL-2 RNA-LNP–treated animals, contributing to a reduced CD8^+^ T–to–T_reg_ cell ratio compared with predose (Fig. 5D). By contrast, T_reg_ cell proportions remained unaltered in animals treated with Alb–IL-2var RNA-LNP (60 μg/kg) and were only slightly elevated in the 180 μg/kg group. Consequently, mean CD8^+^ T–to–T_reg_ cell ratios were consistently higher in the Alb–IL-2var RNA-LNP groups than in the control group (Fig. 5E), further substantiating the potential of Alb–IL-2var RNA-LNP to selectively enhance CD8^+^ T cell expansion. In conclusion, Alb–IL-2var RNA-LNP exhibits a pharmacological profile in NHPs that resembles our observations in mouse models, where it was associated with potent antitumoral activity in conjunction with cancer vaccines, CPI, and LRT.

### Alb–IL-2var shows a manageable nonclinical tolerability profile

To support first-in-human development, Alb–IL-2var RNA-LNP was evaluated in repeat-dose GLP and non-GLP safety studies in mice and cynomolgus monkeys, including clinical observations, clinical pathology, and histopathology assessments (table S1). Across species, Alb–IL-2var RNA-LNP was tolerated at pharmacologically active dose levels, and the observed findings were largely consistent with its mechanism of action and hepatic LNP delivery.

In mice, once-weekly intravenous administration up to 3 μg per animal (∼150 μg/kg) was generally well tolerated. Treatment-related findings comprised transient reductions in circulating leukocytes, cytokine induction, lymphoid hyperplasia, and Kupffer cell hypertrophy/hyperplasia. These changes were reversible after a 3-week recovery period and were not accompanied by histopathological evidence of overt organ toxicity. Mild and transient liver enzyme elevations were observed in some studies, but, together with the Kupffer cell findings, were interpreted as reflecting hepatic uptake/transfection and local cytokine production rather than adverse hepatocellular injury.

In cynomolgus monkeys, repeat dosing up to 180 μg/kg was also generally well tolerated, with no test item–related mortality, no vascular leak-like clinical signs, no clinically meaningful effects on body weight or food consumption, and no clinically relevant liver toxicity. Transient postdose lymphocyte changes were observed, in line with the expected pharmacodynamic activity of IL-2 pathway stimulation.

Treatment immunogenicity was also assessed. In mice, antidrug antibodies against Alb–IL-2 and Alb–IL-2var fusion proteins were detected, as expected for human cytokine-containing fusion proteins in this species, and anti-PEG antibodies were also observed, without an apparent reduction in repeat liver mRNA translation. In cynomolgus monkeys, no Alb–IL-2var–specific antidrug antibodies were detected, although anti-PEG antibodies occurred in some animals, consistent with prior reports for PEG-containing formulations (*54*).

Exploratory combination tolerability studies with anti–PD-1 or RNA-LPX did not reveal new or qualitatively distinct safety signals at clinically relevant dose levels. Together, these data indicate that Alb–IL-2var RNA-LNP has a manageable and largely mechanism-consistent nonclinical tolerability profile that supported clinical evaluation.

## DISCUSSION

Here, we report the preclinical development of Alb–IL-2var RNA-LNP, an RNA-encoded LNP-formulated IL-2 variant with improved pharmacokinetic and pharmacodynamic properties. Alb–IL-2var combines previous advances in IL-2 engineering into a single, clinically translatable format. IL-2var was engineered to enhance IL-2Rβ binding while reducing, but not eliminating, IL-2Rα binding. This design aims to favor expansion of antigen-experienced CD8^+^ T cells (CD25^high^CD122^high^) over T_reg_ cells (CD25^very high^CD122^int^). When delivered as an RNA-encoded albumin fusion protein via LNP, IL-2var achieved prolonged systemic exposure with detectable serum levels for more than 72 hours and promoted selective CD8^+^ T cell expansion with limited T_reg_ cell activation in both mice and NHPs, resulting in a consistently elevated CD8^+^ T–to–T_reg_ cell ratio while also showing a manageable nonclinical tolerability profile. We demonstrated that Alb–IL-2var RNA-LNP outperforms Alb–IL-2 RNA-LNP in enhancing the efficacy of RNA-LPX-based cancer vaccines in several mouse tumor models. In both immunogenic (CT26) and poorly immunogenic (B16F10) settings, Alb–IL-2var RNA-LNP amplified vaccine-induced, tumor-specific CD8^+^ T cell responses while maintaining low T_reg_ cell levels, leading to improved CD8^+^ T–to–T_reg_ cell ratios, complete tumor regression in CT26 tumor-bearing mice, and significantly prolonged survival in B16F10 tumor-bearing mice. It increased intratumoral TCF1^+^ PD-1^+^ antigen-specific T cells, the primary responding cell type after anti–PD-1 therapy and a population known to play a key role in the therapeutic activity of cancer vaccines (*48*, *49*), reduced the proportion of potentially exhausted TOX^+^ PD-1^+^ cells (*38*, *39*), and enhanced the efficacy of an anti–PD-L1 antibody across multiple therapeutic contexts. Unlike non-alpha IL-2 variants that completely lack IL-2Rα binding and fail to activate antigen-specific T cells, Alb–IL-2var retains sufficient IL-2Rα binding to support expansion of activated tumor antigen–specific CD8^+^ T cells. In murine models, Alb–IL-2var RNA-LNP synergized with anti–PD-L1 and, in triple combination with LRT or a cancer vaccine, led to robust tumor control, increased survival, and improved infiltration of effector T cells while limiting T_reg_ cell expansion. Together, these findings support the developmental potential of Alb–IL-2var RNA-LNP as a next-generation immunotherapeutic designed to overcome key limitations of conventional IL-2–based therapies.

We used the RNA-LNP delivery platform to combine the distinct receptor binding profile of Alb–IL-2var with this platform’s favorable pharmacokinetics, characterized by a delayed increase in systemic exposure and an extended half-life. This pharmacokinetic profile differs from most previously reported half-life extended IL-2 variants, which are protein-based drugs administered in their active form. As a result, their pharmacokinetics are characterized by a distinct and short period of peak serum concentration followed by a continuous decline (*55*–*58*). An exception is bempegaldesleukin, which follows kinetics similar to Alb–IL-2var RNA-LNP, with maximal serum concentration of its bioactive species being reached 1 to 2 days after administration of the inactive prodrug in mice (*59*).

Although our data describe the pharmacology and combination activity of Alb–IL-2var RNA-LNP, the mechanistic analyses remain preliminary. We did not comprehensively characterize changes in intratumoral dendritic cells and macrophages, T_reg_ activation state and function, or the cytotoxicity, phenotypic heterogeneity, and long-term persistence of expanded tumor-reactive CD8^+^ T cells. However, several findings suggest favorable remodeling of the CD8^+^ T cell compartment: Alb–IL-2var RNA-LNP increased TCF1^+^PD-1^+^ and reduced TOX^+^PD-1^+^ antigen-specific CD8^+^ T cells, consistent with a less terminally exhausted phenotype. In blood, CD127^+^KLRG1^−^ memory-precursor frequencies were preserved, and CD127^+^KLRG1^+^ tumor antigen-specific CD8^+^ T cells increased, a subset reported to contribute to memory (*37*). Together with the expansion of antigen-specific CD8^+^ T cells, this pattern is consistent with maintained or increased longer-term immune potential.

Protein-based IL-2 variants with modulated IL-2Rα binding have long dominated the space of engineered IL-2 therapeutics but have failed to demonstrate meaningful clinical activity (*60*). Some variants, such as NL-201 (*15*), THOR-707 (*61*), or MDNA11 (*18*), completely abrogate IL-2Rα binding, whereas bempegaldesleukin in its bioactive forms exhibits some residual binding to IL-2Rα (*62*). Tumor antigen–specific CD8^+^ T cells express IL-2Rα, and affinity to IL-2Rα is essential for IL-2 to activate antigen-specific CD8^+^ T cells in chronic viral infection (*42*) and tumor models (*43*, *63*, *64*). Eliminating the interaction of IL-2 with IL-2Rα can abolish synergism with PD-1 CPI (*42*). We observed that Alb–IL-2var RNA-LNP, with its retained IL-2Rα affinity, is able to expand both vaccine-induced CD8^+^ T cells that up-regulate IL-2Rα upon priming (*23*, *65*), as well as tumor-infiltrating CD8^+^ T cells specific for cancer neoantigens including the TCF1^+^PD-1^+^ “stem-like” subset (*48*, *49*), while reducing the fraction of potentially exhausted TOX^+^PD-1^+^ tumor-specific CD8^+^ T cells (*38*, *39*). This effect is associated with a pronounced synergy with PD-1/PD-L1 CPI, further supporting that Alb–IL-2var RNA-LNP has sufficient residual IL-2Rα affinity to stimulate tumor-reactive CD8^+^ T cells.

While selective enhancement of IL-2Rβγ signaling without reducing IL-2Rα binding has been proposed as an alternative strategy (*13*), our earlier comparative studies (fig. S5) indicated that combining enhanced IL-2Rβγ binding with partial IL-2Rα attenuation gave the most favorable balance between expansion of antigen-specific CD8^+^ T cells and avoidance of T_reg_ cell expansion. This balance also helps explain model-dependent differences between Alb–IL-2var RNA-LNP and Alb–IL-2 RNA-LNP in vaccine combinations. In CT26, where preexisting gp70-specific T cell responses are present (*66*, *67*), both agents similarly expanded circulating antigen-specific CD8^+^ T cells, but Alb–IL-2var showed better efficacy, likely because it drove less T_reg_ expansion. In B16F10, where Trp-1–specific responses must be primed de novo, Alb–IL-2var, but not Alb–IL-2, enhanced vaccine-induced CD8^+^ T cell expansion, consistent with reduced T_reg_ cell–mediated suppression during priming (*68*). Further supporting the notion that IL-2 mediated systemic T_reg_ cell expansion can impair vaccine-induced CD8^+^ T cell responses, we recently observed that the scheduling of IL-2 relative to vaccine administration heavily affects the expansion of CD8^+^ T cells, which may be partially attributed to T_reg_ cell–mediated suppression when Alb–IL-2 RNA-LNP is added to the vaccine early on. These findings suggest that limiting systemic T_reg_ cell expansion is important for supporting vaccine-induced immunity across priming and subsequent expansion, a situation that is strongly fostered by Alb–IL-2var RNA-LNP.

Together, Alb–IL-2var RNA-LNP could potentially address major limitations of IL-2–based therapies and represents a promising combination partner for conventional cancer therapies such as LRT, cancer vaccines, CPI, and, potentially, adoptive cell therapies. Following the results of this study, Alb–IL-2var RNA-LNP, designated BNT151, entered a first-in-human clinical trial for patients with solid tumors (NCT04455620).

## MATERIALS AND METHODS

### Cell lines

HEK293T/17 cells [American Type Culture Collection (ATCC), CRL-11268] were cultured in DMEM (Thermo Fisher Scientific, 10564011) supplemented with 10% fetal bovine serum (FBS; Sigma-Aldrich, F7524). Expi293F cells (Thermo Fisher Scientific, A14527) were cultured in Expi293 Expression Medium (Thermo Fisher Scientific, A1435101). The CT26 colon carcinoma cell line (ATCC, CRL-2638) was cultured in RPMI 1640 (ATCC, 30-2001) supplemented with 10% FBS. The MC38 colon carcinoma cell line (Leiden University Medical Center, The Netherlands) was cultured in RPMI 1640 supplemented with 10% FBS, 2 mM l-glutamine (Thermo Fisher Scientific, 25030081). The B16F10 melanoma cell line (ATCC, CRL-6475) was cultured in DMEM (Thermo Fisher Scientific, D5030) supplemented with 10% FBS (not heat inactivated). The HPV16^+^ TC-1 lung carcinoma cell line (provided by T.-C. Wu, Johns Hopkins University) was cultured in IMDM (Thermo Fisher Scientific, 21980032) supplemented with 8% FBS (heat inactivated), 1 mM sodium pyruvate (Thermo Fisher Scientific, 11360070), and 0.1 mM nonessential amino acids (Thermo Fisher Scientific, 11140050). The cell lines were passaged at least three times and were harvested for in vivo experiments when growing in log phase.

### Animals

C57BL/6 mice were purchased from Envigo, and BALB/c mice were purchased from Janvier Labs. Age-matched (6 to 13 weeks) female animals were used in all experiments. Experimental protocols were approved by the local welfare committee Landesuntersuchungsamt Rheinland-Pfalz (NTP-IDs 00019862, 00022142, 00036296, and 00038090) and conducted according to Federation of European Laboratory Animal Science Associations recommendations. Study execution and housing complied with the German Animal Welfare Act and Directive 2010/63/EU. All mice were kept in accordance with federal and state policies on animal research at BioNTech SE. Studies in captive-bred, female cynomolgus monkeys (*Macaca fascicularis*) were performed by LPT Laboratory of Pharmacology and Toxicology GmbH & Co. KG (Hamburg, Germany) in accordance with federal and state policies on animal research.

### Construct design and in vitro translation

Templates for in vitro transcription were obtained by cloning target sequences into plasmids derived from the pCMV-Script-Vector (Stratagene) (*69*, *70*). All vectors were pharmacologically optimized for RNA stability and protein translation (*70*) and encoded a T7 promoter, a derivative of 5′ human α-globin–untranslated region (UTR), a 3′ FI element [where F is a 136-nucleotide (nt)–long 3′UTR fragment of N-terminal enhancer of split mRNA and I is a 142-nt-long fragment of mitochondrially encoded 12*S* RNA both identified in *Homo sapiens*] (*71*) and a poly(A) tail of 100 nt, with a linker after 70 nt.

Plasmids for transcription of antigen-encoding RNA feature a signal sequence for routing to the endoplasmic reticulum and the transmembrane and cytoplasmic domains of major histocompatibility complex (MHC) class I for improved presentation of MHC class I and II epitopes (*69*). Target sequence encoded one of the following epitopes: gp70 (*66*), Trp-1, or the full-length HPV16 E7 protein (*72*). The gp70 vector encoded the H-2L^d^–restricted peptide antigen AH1_423-431_ derived from Moloney murine leukemia virus envelope gp70 with an amino acid substitution at position 5 (V427A; AH1-A5) (*73*). The Trp-1 construct encoded the H-2D^b^–restricted epitope Trp-1_455-463_ derived from the murine Trp-1 protein, with an amino acid substitution at position 9 (A463M) (*41*). The E7 construct encoded full-length E7 protein of HPV-16. For the irrelevant RNA control, the empty vector was used.

The Alb–IL-2 construct contained the original sequence of human IL-2 (NP_000577.2; NCBI protein resource; www.ncbi.nlm.nih.gov/protein/). The Alb–IL-2_s construct contained the sequence of IL-2 with the following amino acid substitutions: L80F, R81D, L85V, I86V, and I92F (*13*). The Alb–IL-2_M2-encoding construct contained the sequence of IL-2 with the following amino acid substitutions: K43E and E61K (*10*). The mutation set in the Alb–IL-2_M2s construct combined the mutations of IL-2_s and IL-2_M2 constructs: K43E, E61K, L80F, R81D, L85V, I86V, and I92F. The Alb–IL-2var construct contained the following substitutions in the human IL-2 sequence: K43E, E61K, Q74H, L80F, R81E, L85V, I92F. Cytokine- and serum Alb–encoding sequences originate from *H. sapiens*, and no changes in the resulting amino acid sequences were introduced, except for the intended mutations in the IL-2 variants described above. For cytokine constructs, the IL-2 variant was added to the C terminus of Alb, and encoded proteins were equipped with an N-terminal signal peptide (SP). For fusion proteins, only the SP of the N-terminal moiety was maintained; for further moieties, only the mature protein without SP was encoded. A stop codon was introduced for the C-terminal moiety only. Different protein moieties in the cytokine and Alb fusion constructs were connected by a 30-nt-long linker sequence encoding glycine and serine residues. RNA that only encoded Alb was used as control.

### In vitro transcription

The DNA was linearized, spectrophotometrically quantified, and subjected to in vitro transcription with T7 RNA polymerase (Thermo Fisher Scientific) as previously described (*72*). The transcription of RiboCytokine, Alb, and luciferase RNAs involved the use of modified nucleosides. Resulting RNAs were equipped with a CleanCap-structure (TriLink) as previously described (*70*, *72*). Double-stranded RNA molecules were depleted by cellulose purification based on a previously reported procedure (*74*). Purified RNA was eluted in H_2_O and stored at −80°C until further use. In vitro transcription of all described RNA constructs was carried out at BioNTech.

### Preparation of RNA-LNPs and RNA-LPX

RiboCytokine, Alb, and luciferase RNAs were encapsulated within LNPs (Genevant Sciences Corporation, except for the experiment shown in fig. S5, in which an earlier in-house LNP candidate was used), and the resulting RNA-LNP formulations were stored at −60° to −80°C. For injection, RNA-LNP aliquots were thawed at ambient temperature and diluted to 100 μg/ml with phosphate-buffered saline (PBS; Thermo Fisher Scientific, 14190144) and filtered into a fresh container. Filtered LNPs were further diluted with PBS to the final concentration.

RNA-LPX formulation was performed according to proprietary protocols, derived from extensive internal formulation development activities (*29*)_._ Briefly, liposomes were used to form RNA-LPX complexes of a negative net charge. The liposomes were manufactured using an adapted proprietary protocol (*75*) from the cationic synthetic lipid (R)-*N*,*N*,*N* trimethyl-2-3-dioleyloxy-1-propanaminium chloride (Merck and Cie) and the phospholipid 1,2-dioleoyl-*sn*-glycero-3-phosphoethanolamine phospholipid (Corden Pharma).

### Bioluminescence imaging

Bioluminescence imaging was performed using the Xenogen IVIS Spectrum imaging system (Caliper Life Sciences). Mice injected with luciferase-encoding RNA-LNPs were injected intraperitoneally with 250 μl of an aqueous solution containing 1.6 mg of d-luciferin (PerkinElmer, 122799-10). Photons emitted from live animals or extracted organs were measured 10 min later. Regions of interests were quantified using IVIS Living Image 4.5.5.

### Pharmacokinetic assessment

The serum concentration profile of Alb–IL-2var protein in mice was established after a single intravenous dose of 10 μg of Alb–IL-2var RNA-LNP per animal. Alb-encoding RNA formulated as LNP was used as a control. Blood was collected at 1, 4, 24, 48, 72, 96, 116, 140, and 164 hours after injection. For the analysis of the pharmacokinetic profile of Alb–IL-2var in NHPs, female cynomolgus monkeys were administered with 50 μg of Alb–IL-2var RNA-LNP per animal (on average, 19.45 μg of IL-2var/kg) on study days 1 and 8. Serum for bioanalysis was collected at 6, 12, 24, 48, 72, and 144 hours after each dosing. Serum concentrations of Alb–IL-2var were determined using a custom-developed V-PLEX human Alb–IL-2var kit (Meso Scale Diagnostics LLC), according to the manufacturer’s protocol. Recombinant Alb–IL-2var was used as a standard. The plate was analyzed on a MESO QuickPlex SQ 120 imager (Meso Scale Diagnostics).

### Generation of cytokine-containing supernatants

HEK293T/17 cells were transfected with RNA using Lipofectamine MessengerMax transfection reagent (Thermo Fisher Scientific, LMRNA001) according to the manufacturer’s protocol. Supernatants were collected 1 day after the transfection, centrifuged to pellet residual cells, and stored at −20°C until use.

### STAT5 phosphorylation assay

Peripheral blood mononuclear cells (PBMCs) were isolated from human whole blood (University Hospital Mainz, Germany) by density gradient centrifugation (VWR, Ficoll-Paque PLUS medium, 17-5446-02). PBMCs were rested in X-VIVO 15 serum-free hematopoietic cell medium (Lonza, BE02-060Q) for 1 hour. CD8^+^ T cells were isolated from PBMCs using CD8 microbeads (Miltenyi, 130-045-201) and LS columns (Miltenyi, 130-042-401) and electroporated with RNA encoding IL-2Rα (15 μg). Electroporated CD8^+^ T cells were incubated overnight in IMDM medium (Gibco, 12440-053) with 5% human serum (ONE LAMBDA, A Thermo Fisher Scientific Brand, A25761). For assessment of STAT5 phosphorylation, rested PBMCs or electroporated CD8^+^ T cells were incubated with serial dilutions of cytokine-containing HEK293T/17-cell supernatants for 10 min. STAT5 phosphorylation was analyzed by flow cytometry. CD4, CD8, CD25, and FoxP3 were used as lineage markers to identify cytotoxic T cells (CD8^+^) and T_reg_ cells (CD4^+^CD25^+^FoxP3^+^). For determination of EC_50_ values, STAT5 phosphorylation was displayed as functions of cytokine-containing supernatant concentration using GraphPad Prism. Data were fitted and EC_50_ values derived using the log(agonist) versus response-variable slope (four parameters) equation. On some occasions, the potency to stimulate STAT5 phosphorylation was calculated as the reciprocal EC_50_ value. If no EC_50_ could be determined, then an arbitrary potency of half the lower limit of quantification (reciprocal of the highest cytokine-containing supernatant concentration) was used.

### Production of recombinant IL-2 protein and variants

Genes encoding the different IL-2 variants with an N-terminal His_6_-tag were synthesized and cloned into pTwist_CMV_BetaGlobin_WPRE_Neo expression vector (Twist Bioscience). Protein production was conducted using ExpiFectamine 293 transfection kit (Thermo Fisher Scientific, A14524) following the manufacturer’s instruction. Precultured Expi293F cells were adjusted to a density of 3 × 10^6^ cells/ml in a volume of 25 ml. Plasmid (30 μg) was diluted in 1.5 ml of Opti-MEM (Thermo Fisher Scientific, 31985070). ExpiFectamine (80 μl) in 1.5 ml of Opti-MEM was incubated for 5 min at room temperature (RT), and the mixture was added to the plasmid solution and incubated for 20 min at RT. The solution was slowly transferred to the cells. Cells were cultured for 20 hours at 37°C, 8% CO_2_ at 120 rpm. Afterward, a mixture of 150 μl of enhancer 1 and 1.5 ml of enhancer 2 was added to the cells. After 4 days of additional culture, cell supernatants were collected and His-tagged proteins were purified via the Capturem His-Tagged Purification Maxiprep Kit (TaKaRa, 635713). Elution fractions were pooled, dialyzed against PBS, and stored at 4°C.

### Affinity measurements of recombinant IL-2 protein and variants

Affinity measurements of recombinant IL-2 protein and variants to recombinant IL-2Rα or IL-2Rβ were performed using an Octet RED device (FortéBio) at RT. Ni–nitrilotriacetic acid sensors (Sartorius Lab Instruments, 18-5101) were equilibrated in 200 μl of PBS supplemented with 0.05% Tween 20 (Merck Millipore, 655205) for 10 min. Samples were measured in a black 96-well microtiter plate in a volume of 200 μl. After recording an initial baseline in Octet Kinetics Buffer (Sartorius Lab Instruments, 18-1105) for 60 s, loading of sensors with IL-2 proteins (100 μg/ml) was performed for 120 s. Subsequently, a baseline was measured in Octet Kinetics Buffer for 60 s. Binding of captured IL-2 protein or variants toward IL-2Rα (Novoprotein, CJ78) with concentrations from 9.38 to 300 nM or IL-2Rβ (Novoprotein, CJ82) with concentrations from 6.25 to 200 nM was measured over an association period for 300 s followed by a dissociation period of 420 s.

Data were acquired on Octet RED system with the program Octet Data Acquisition 9.0 and analyzed using Data Analysis Software 9.0 (FortéBio). Binding kinetics were calculated from reference subtracted data using a global 1:1 binding model with coefficient of determination (*R*^2^) value greater than 0.97.

### Mouse tumor models and treatment

Tumor cell lines were harvested from a culture growing in log phase (∼90% viability) and counted. CT26 cells were subcutaneously injected into the upper flank of female BALB/c mice (5 × 10^5^ cells per mouse in 100 μl of PBS). B16F10 cells were subcutaneously injected into the upper flank of female C57BL/6 mice (3 × 10^5^ cells per mouse in 100 μl of PBS). MC38 cells were subcutaneously injected into the upper flank of female C57BL/6 mice at 5 × 10^5^ (Fig. 3E) or 7.5 × 10^5^ (all other studies) cells per mouse in 100 μl of PBS. TC-1 cells were subcutaneously injected into the upper flank of female C57BL/6 mice (1 × 10^5^ cells per mouse in 100 μl of PBS).

Mice were stratified according to tumor size using the ANOVA-P method (Daniel’s XL Toolbox) immediately before the start of treatment. Group mean or median tumor volumes were calculated. Tumor volumes of euthanized animals were included in the calculations according to the last observation carried forward principle.

CT26 tumor-bearing mice were vaccinated with 20 μg of gp70 RNA-LPX, or irrelevant RNA-LPX as control, in PBS and treated with 3 μg of RiboCytokines (Alb–IL-2var RNA-LNP or Alb–IL-2 RNA-LNP; Alb RNA-LNP as control) in PBS through intravenous injection on days 10, 17, 24, and 31 after tumor inoculation.

B16F10 tumor-bearing mice were vaccinated with 20 μg of Trp-1 RNA-LPX, or irrelevant RNA-LPX as control, in PBS and treated with 3 μg of RiboCytokines (Alb–IL-2var RNA-LNP or Alb–IL-2 RNA-LNP; Alb RNA-LNP as control) in PBS through intravenous injection on days 8, 15, 22, 29, and 36 after tumor inoculation.

MC38 tumor-bearing mice were treated with 3 μg of RiboCytokine (Alb–IL-2var RNA-LNP; Alb RNA-LNP as control) in PBS through intravenous injection on days 17, 24, 32, and 39 after tumor inoculation and intraperitoneally injected with 100 μg of αPD-L1 (InvivoGen, hpdl1-mab9) or isotype control antibodies (day 17: 200 μg antibody) on the same days as RiboCytokine treatments. For the experiment involving LRT, LRT was performed on day 19, and mice were treated with 3 μg of RiboCytokines (Alb–IL-2var RNA-LNP; Alb RNA-LNP as control) in PBS through intravenous injection on days 26, 33, 41, and 47 after tumor inoculation and intraperitoneally injected with 200 μg of αPD-L1 or isotype control antibodies on the same days as RiboCytokine treatments. For LRT, a custom-made lead shield containing 1.5-cm-wide circular cavities positioned above the tumors was used to facilitate the LRT of tumors while shielding the body of the animal. Mice were irradiated with a single dose of 12 gray using an X-RAD 320 (PrecisionX-Ray Instruments). For analysis of tumor-infiltrating immune cells, MC38 tumor-bearing mice received a single intravenous injection of 3 μg of RiboCytokines (Alb–IL-2var RNA-LNP; Alb RNA-LNP as control) in PBS on day 19 after tumor inoculation, before the mice were euthanized and tumors were harvested for analysis on day 24.

TC-1 tumor-bearing mice were vaccinated with 40 μg E7 of RNA-LPX, or irrelevant RNA-LPX as control, in PBS through intravenous injection on day 13 after tumor inoculation. The mice were treated with 3 μg of RiboCytokine (Alb–IL-2var RNA-LNP; Alb RNA-LNP as control) in PBS through intravenous injection on days 18, 25, and 32 after tumor inoculation and, in addition, received a total of seven weekly intraperitoneal injections of 100 μg of αPD-L1 or isotype control antibodies (day 18, 200 μg of antibody), starting on day 18. For analysis of tumor-infiltrating immune cells, TC-1 tumor-bearing mice were vaccinated with 40 μg of E7 RNA-LPX, or irrelevant RNA-LPX as control, in PBS through intravenous injection on day 13 after tumor inoculation. The mice received a single intravenous treatment with 3 μg of RiboCytokines and 200 μg of αPD-L1 or isotype control antibody on day 18, before the mice were euthanized and tumors were harvested for analysis on day 24.

### Pharmacodynamic studies in NHPs

To evaluate lymphocyte expansion, including CD8^+^ T cells and T_reg_ cells, female cynomolgus monkeys were administered Alb–IL-2var RNA-LNP (60 or 180 μg/kg), or empty LNPs (lipid dose adjusted to 120 μg RNA/kg) as a control, on study days 1 and 22. Lymphocyte counts were determined by hematocytometry on days 8 and 29, whereas CD8^+^ T cells, NK cells, and T_reg_ cells were analyzed by flow cytometry on day 8.

### Flow cytometry

Blood was collected from mice via puncture of the facial vein. Tumor single-cell suspensions were prepared by enzymatic digestion using the mouse tumor dissociation kit (Miltenyi Biotec, 130-096-730) and the gentleMACS dissociator (Miltenyi Biotec) according to the manufacturer’s protocol.

Dead cells in mouse tumor samples were stained for 15 min at 2° to 8°C using eF506 (eBioscience, 65-0866-18), eF780 (eBioscience, 65-0865-14), or Fixable Yellow Dead Cell Stain (Thermo Fisher Scientific, L34959). Staining of extracellular markers was carried out at 2° to 8°C for 30 min. Erythrocytes were subsequently removed from blood samples by 8 min incubation in BD FACS Lysing Solution (BD Biosciences, 349202) at RT. For intranuclear staining of mouse samples, cells were fixed and permeabilized for 30 min at 2° to 8°C using the Foxp3/Transcription Factor Staining Buffer Set (eBioscience, 00-5523-00) followed by intracellular staining for 30 min at 2° to 8°C. For intracellular staining of cynomolgus monkey samples, cells were fixed, permeabilized, and stained using the Human FoxP3 Buffer Set (BD Biosciences, 560098). For assessment of STAT5 phosphorylation, cells were stained with fixable viability dye (eBioscience, 65-0865-18), followed by fixation with 2% formaldehyde (Carl Roth, P087.1) for 10 min at 2° to 8°C and permeabilization with methanol (Carl Roth, 7342.2) for 30 to 60 min at 2° to 8°C followed by staining of extra- and intracellular markers for 30 min at 2° to 8°C.

The following antibodies were used: anti-mouse CD4 (clone RM4-5; BD Biosciences, 565634; and BioLegend, 100559), CD8 (clone 53-6.7; BD Biosciences, 553031 and 564297), CD8 (clone 5H-10; Invitrogen, MCD0801), CD11c (clone N418; Miltenyi Biotec, 130-102-493), CD11b (clone M1/70; BD Biosciences, 553310), CD25 (clone PC61; BioLegend, 102043; and BD, 553866), CD45 (clone 30-F11; BD Biosciences, 553081, 563053, 564225, and 564279), CD103 (clone 2E7; eBioscience, 12-1031-83), CD127 (clone A7R34; Life Technologies, 1902317), CD206 (clone C068C2; BioLegend, 141719), F4/80 (clone EMR1; BioLegend, 123146), FoxP3 (clone FJK-16s; Invitrogen, 25-5773-82), KLRG1 (clone 2F1; Invitrogen, 4346707), MHC II (clone M5/114.15.2; BD, 742894), PD-1 (clone RMP1-30; BD Biosciences, 1050191; and clone J43; Invitrogen, 1918384), TCF1 (clone C63D9; Cell Signaling Technology, 9066S), TOX (clone REA473; Miltenyi, 5210205027), XCR1 (clone ZET; BioLegend, 148220), and NK1.1 (clone PK136; BD Biosciences, 740853); antihuman CD4 (clone SK3; BD Biosciences, 565997), CD8 (clone SK1; BD Biosciences, 563919), CD25 (clone M-A251; BD Biosciences, 560503), FoxP3 (clone PCH101; Thermo Fisher Scientific, 17-4776-42), FoxP3 (clone 259D/C7; BD Biosciences, 560047), and pY694-STAT5 (clone 47/Stat5; BD Biosciences, 612598). Antigen-specific CD8^+^ T cells were quantified within the live CD45^+^CD4^−^CD8^+^ population using H-2L^d^/gp70_423-431_ (MBL, TB-M521-2) or H2D^b^/Trp-1_455-463_ tetramers (Immunaware, 1824802), as described previously (*29*), or using H2-Kb/N4bp2l2 (MBL, ATINFRRL custom tetramer), H2-D^b^/Adpgk (MBL, TB-5113-2), H2-Kb/Rpl18 (MBL, KILTFDRL custom tetramer), or H2-D^b^/HPV16 E7 (Immudex, 20151005-CD11) multimers.

Data were acquired on a BD FACSCelesta, BD FACSCanto, or a BD FACSymphony A3 flow cytometer (BD Biosciences) and analyzed with FlowJo (FlowJo LLC, BD Biosciences). Cynomolgus monkey samples were measured on a Cytomics FC 500 (Beckmann Coulter) cytometer. For carboxyfluorescein diacetate succinimidyl ester–based proliferation analysis, the generation peaks were fitted, and expansion index values were calculated automatically using the FlowJo proliferation modeling tool.

### Statistics

Unless noted otherwise, the mean is used as the measure of central tendency, and error bars represent the SD. Biological replicates were used in all experiments unless stated otherwise. Student’s unpaired *t* test was performed for comparison of two groups. One-way analysis of variance (ANOVA) with Dunnett’s post hoc test or Kruskal-Wallis test with Dunn’s post hoc test was performed when more than two groups were compared. Statistical difference between Kaplan-Meier survival curves was determined using log-rank test with Bonferroni correction for multiple comparisons. Single outliers were identified by Grubb’s test (α = 0.05) where indicated. All statistical analyses were two sided and performed using GraphPad Prism. Not significant (n.s.), *P* > 0.05; *P ≤ 0.05; **P ≤ 0.01; ***P ≤ 0.005; ****P ≤ 0.0001.
