# Supplementary material for: Messenger RNA delivery of a dual CD25/CD122 affinity-tuned IL-2 variant prolongs exposure and potentiates antitumor T cell immunity
Source: Sci Adv. 2026 Sep 4;12(36):eaeh9215. doi: 10.1126/sciadv.aeh9215 (PMC13544207; doi:10.1126/sciadv.aeh9215)
Supplement: Supplementary file 1 [file sciadv.aeh9215_sm.pdf]

Science Advances
AAAS

Supplementary Materials for

Messenger RNA delivery of a dual CD25/CD122 affinity-tuned IL-2 variant prolongs exposure and potentiates antitumor T cell immunity

Mathias Vormehr *et al.*

Corresponding author: Ugur Sahin, ugur.sahin@biontech.de; Mathias Vormehr, mathias.vormehr@biontech.de

Sci. Adv. **12**, eaeh9215 (2026)
DOI: 10.1126/sciadv.aeh9215

This PDF file includes:

Figs. S1 to S5
Table S1

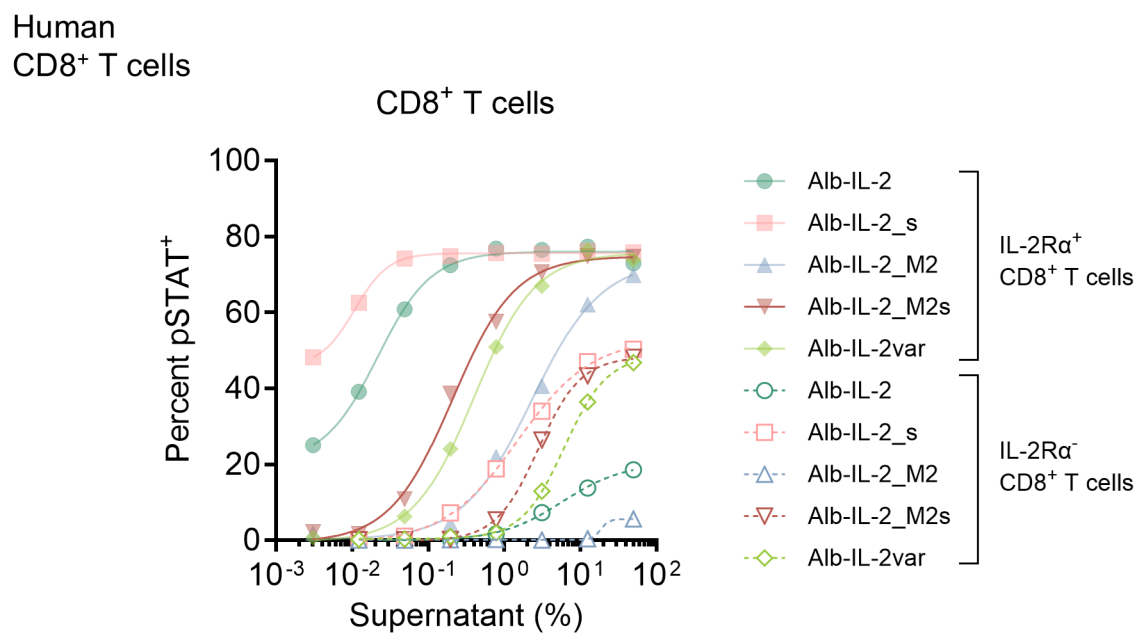

Fig. S1: IL-2 variants differentially stimulate CD8$^+$ T cells in cell culture depending on IL-2Rα expression.

STAT5 phosphorylation in IL-2Rα RNA-electroporated or non-electroporated human peripheral blood CD8$^+$ T cells isolated from PBMCs (n=2 donors) upon incubation with supernatants containing Alb-IL-2 and its variants as determined by flow cytometry.

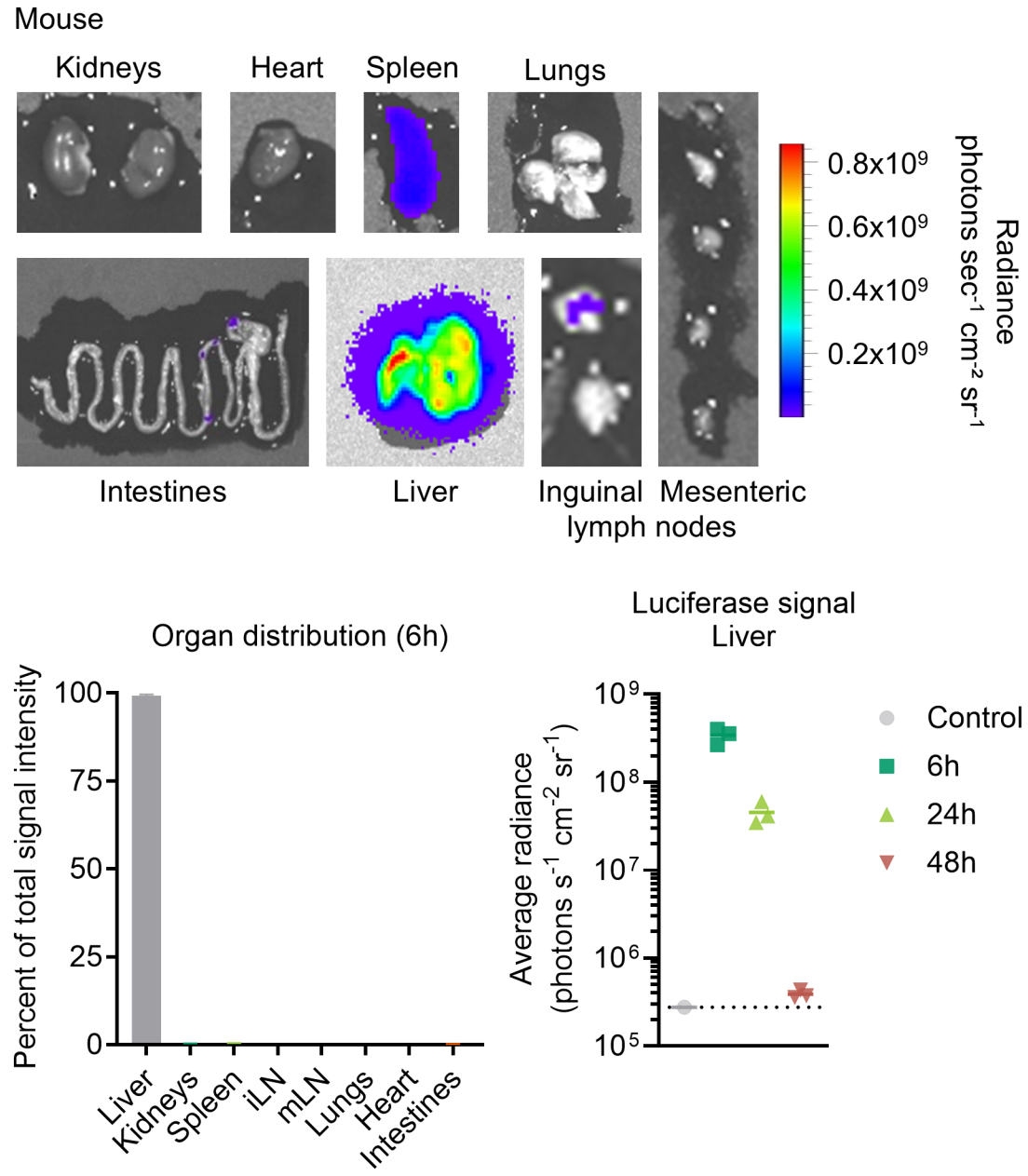

Fig. S2: RNA-LNP-encoded protein is predominantly produced in the liver after intravenous administration in mice.

Representative examples (upper panels) and quantification (lower panels) of bioluminescence in resected organs from BALB/c mice (n=3) 6 hours after intravenous injection of 1 µg firefly luciferase RNA-LNP as described in Fig. 1f. The horizontal dotted line represents the mean of the control group.

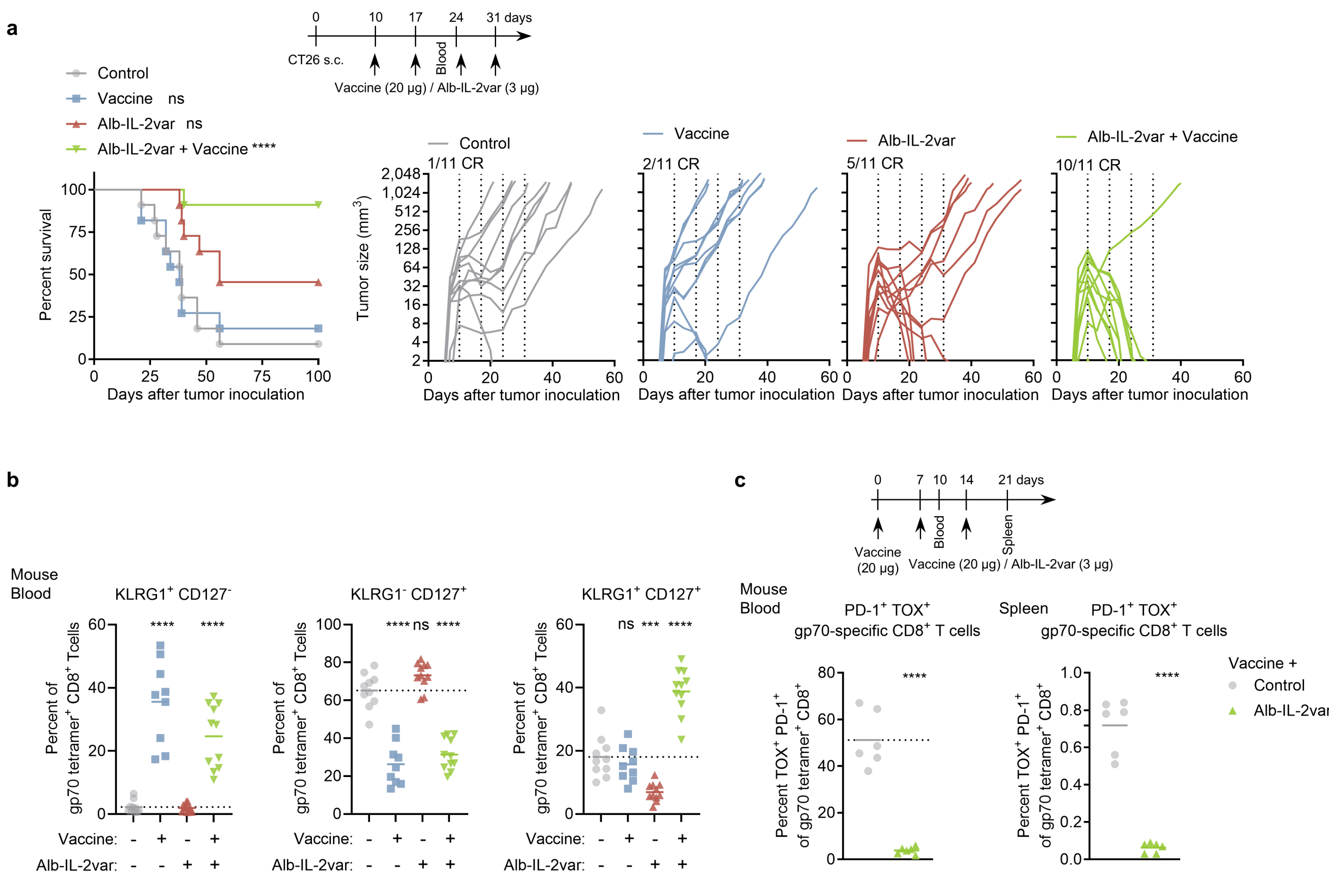

Fig. S3: Combination of RNA vaccine with Alb-IL-2var RNA-LNP is superior to either monotherapy in mice.

a-b, CT26 tumor-bearing BALB/c mice (n=11) treated with gp70 RNA-LPX vaccine (Vaccine) and/or Alb-IL-2var RNA-LNP (Alb-IL-2var) starting on day 10 post tumor inoculation (average tumor volume: 54 mm^3). Combined treatment with irrelevant RNA-LPX and Alb RNA-LNP served as control. (a) Survival and individual tumor growth curves. (b) Frequencies of KLRG1 and CD127 expression among gp70-specific $CD8^+$ T cells in the blood on day 24 (n=9-11). **c,** BALB/c mice (n=6) were vaccinated weekly for three weeks with gp70 RNA-LPX (Vaccine) alone (Control) or together with Alb-IL-2var RNA-LNP (Alb-IL-2var). PD-1^+ TOX^+ gp70 specific T cells on day 10 in blood and on day 21 in spleen, as determined by flow cytometry. Vertical dotted lines represent treatments (a), horizontal dotted lines the means of the control group (b, c). Log-rank test (a, left), two-way ANOVA with Dunnett's multiple comparison test (b), unpaired Student's *t*-test (c). ns=non-significant, $p>0.05$; $*p\leq0.05$; $**p\leq0.01$; $***p\leq0.001$; $****p\leq0.0001$.

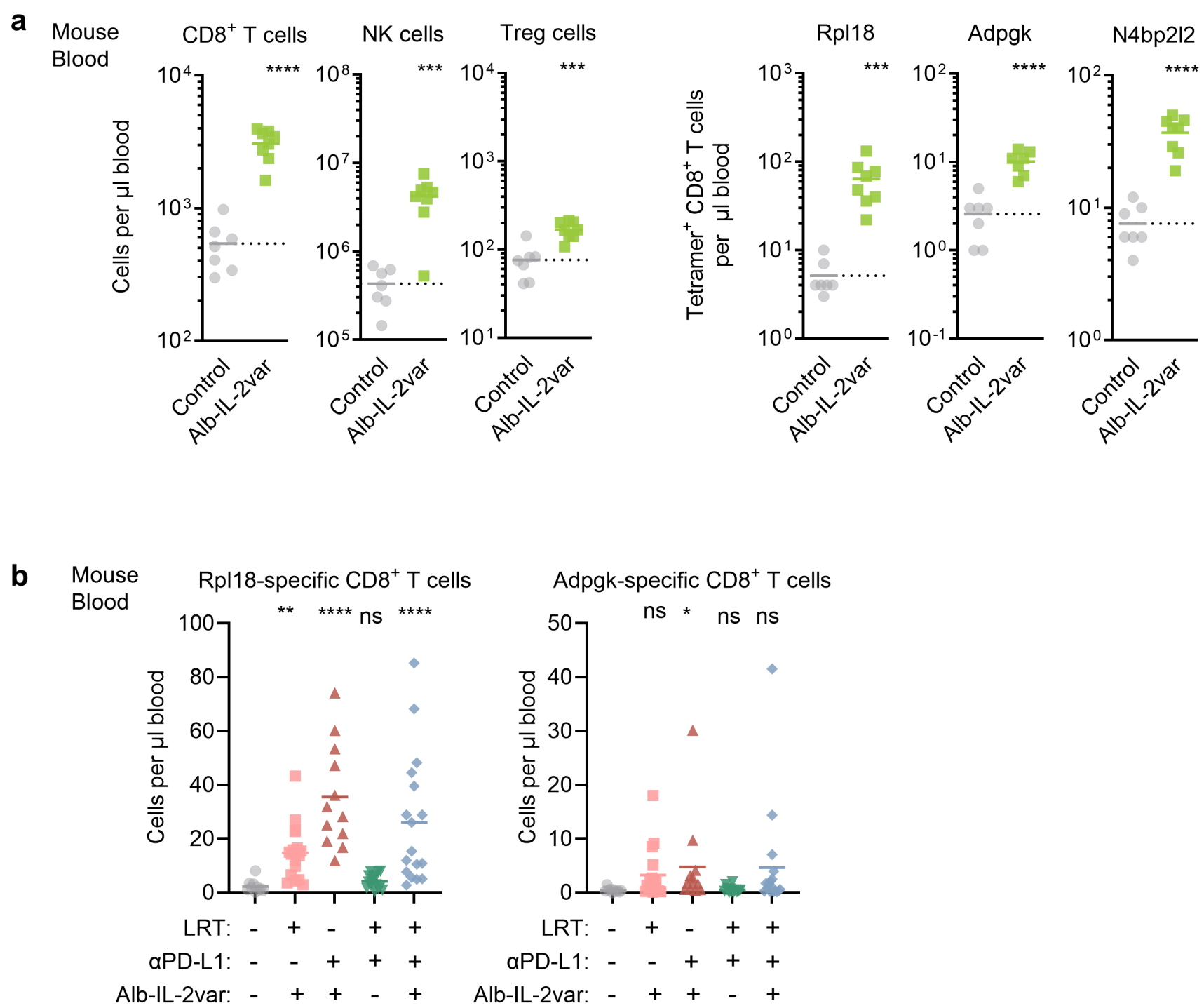

Fig. S4: Alb-IL-2var RNA-LNP drives expansion of tumor-antigen-specific CD8$^+$ T cells in mice.

a, MC38 tumor-bearing C57BL/6 mice (n=7–8) were treated with Alb-IL-2var RNA-LNP (Alb-IL-2var) starting on day 18 post tumor inoculation as shown in Fig. 3a. Alb RNA-LNP served as a control. CD8$^+$ T cells, NK cells, and Treg cells as well as neoantigen-specific CD8$^+$ T cells in blood on day 24, as determined by flow cytometry. Horizontal dotted lines represent the means of the control group. **b,** Neoantigen-specific CD8$^+$ T cells in the blood of MC38 tumor-bearing C57BL/6 mice (n=10-17) treated with LRT (12 Gy), Alb-IL-2var RNA-LNP and/or αPD-L1 as determined by flow cytometry and as shown in Fig. 3g. Alb RNA-LNP and isotype served as controls. Unpaired Student's *t*-test (a), Kruskal-Wallis test with Dunn's multiple comparison test (b). ns=non-significant, p>0.05; *p≤0.05; **p≤0.01; ***p≤0.001; ****p≤0.0001.

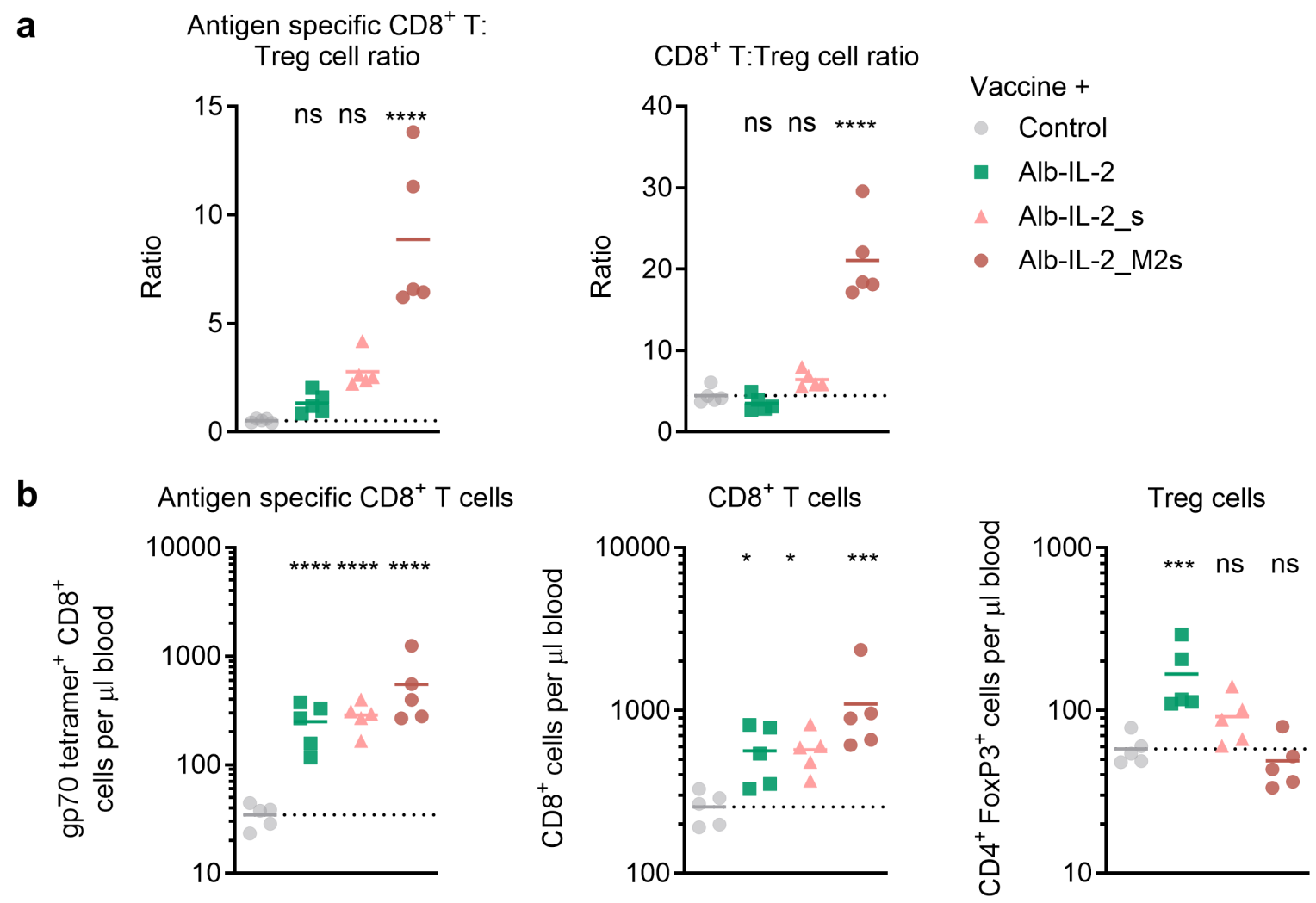

Fig. S5: Combined enhancement of IL-2Rβγ signaling with partial attenuation of IL-2Rα binding improves the antigen-specific CD8+ T-cell-to-Treg ratio over IL-2Rβγ enhancement alone.

a-b, BALB/c mice (n=5) were treated with 20 µg gp70 RNA-LPX vaccine (Vaccine) on days 0, 7, and 14 as well as Alb-IL-2 RNA-LNP (Alb-IL-2), Alb-IL-2_s RNA-LNP (Alb-IL-2_s), or Alb-IL-2_M2s RNA-LNP (Alb-IL-2_M2s) on days 3, 10, and 14. Alb RNA-LNP served as the control. gp70-specific $CD8^+$ T-to-Treg-cell ratio or $CD8^+$ T-to-Treg-cell ratio (a), and gp70-specific $CD8^+$ T cells, $CD8^+$ T cells, and Treg cells (b) in blood 21 days after the first treatment, as determined by flow cytometry. One-way ANOVA with Dunnett's multiple comparison test (a), lognormal one-way ANOVA with Dunnett's multiple comparison test (b). ns=non-significant, $p>0.05$; *$p\leq0.05$; **$p\leq0.01$; ***$p\leq0.001$; ****$p\leq0.0001$.

Tab. S1: Studies on toxicology of Alb-IL-2var alone and in various combinations in mice and cynomolgus monkeys.

Study type	GLP status	Species	Therapy type	Test item and dose per animal	Administration schedule	Observations
Repeated-dose toxicity study	GLP	Mice C57BL/6	Monotherapy	1 or 3 µg Alb-IL-2var	Day 1, 8, 15, 22	Tolerated.<br>Findings related to expected IL-2 pharmacology:<br>Hematology: Transient decreases in circulating white blood cells, lymphocytes, and monocytes. Increased size, weight, cellularity of lymphoid organs.<br>Cytokine/chemokine analysis: Transient induction of IP-10, IFNγ, and IL-6.<br>Histopathology: Lymphoid hyperplasia in spleen, extramedullary hematopoiesis, and Kupffer cell hypertrophy/hyperplasia. Findings largely resolved after three-week recovery period, interpreted as immune activation rather than organ toxicity.<br>Liver-related findings: Kupffer cell hypertrophy/hyperplasia, mild, transient liver enzyme elevations, interpreted as adaptive changes linked to liver transfection and local cytokine production. Severity limited, changes had resolved by end of recovery phase.
Anti-drug immunogenicity study	Non-GLP, exploratory	Mice C57BL/6	Monotherapy	3 µg Alb-IL-2var	Day 1, 8, 15, or Day 1, 15, 29, or Day 1, 22, 50	Anti-Alb-IL-2var protein ADA: IgM responses increased slightly after first dosing, remaining stable or decreasing. IgG responses increased more clearly and steadily until day 21, remaining elevated.<br>Anti-PEG ADA: IgM responses increased slightly after first dosing, then decreasing to baseline. IgG responses hardly detectable.
Anti-drug immunogenicity study	Non-GLP, exploratory	Mice BALB/c	NA	1 µg Luciferase, 1 µg Thy1.1, 2 µg Alb-	Day 0, 7, 14	Anti-PEG ADA: IgM responses slightly increased after first and second dosing. IgG responses hardly detectable.<br>Luciferase signal in liver: No significant difference between Luciferase signal 24 h after first, second and third dose.<br>Thy1.1 signal in hepatocytes: All measured hepatocytes are Thy1.1^{+} 6 h after first and third dose.
Efficacy study	Non-GLP, exploratory	Mice BALB/c	Combination therapy	3 µg Alb-IL-2var + 20 µg RNA-LPX	Day 10, 17, 24, 31	Tolerated.<br>Body weight: Moderate maximal loss of -10.2% in the combination group vs. -3.9% for Alb-IL-2var alone on day 13 as compared to day 0. Mice receiving Alb + irrelevant RNA-LPX control achieved a weight gain of +2.3% by day 13.<br>No obvious behavioral abnormalities or signs of distress.
Anti-PD-1 co-treatment tolerability study	Non-GLP, exploratory	Mice C57BL/6	Combination therapy	0.5 µg or 3 µg Alb-IL-2var + 200 µg αPD-1	Day 1, 8, 15	Addition of PD-1 blockade to the treatment with Alb-IL-2var did not lead to any toxicities (weight loss, clinical chemistry, hematology) beyond Alb-IL-2var monotherapy. Reduction of Alb-IL-2var dose reduced or fully eliminated toxicities observed with higher doses of Alb-IL-2var, independent from PD-1 blockade.
Tolerability study	Non-GLP	Cynomolgus monkeys	Combination therapy	20 or 50 µg Alb-IL-2var + 50 µg RNA-LPX	Day 1, 22	Tolerated. No systemic intolerance reactions. One death on day 23, not test-item related.<br>No influence on body weight, food consumption.<br>Electrocardiography and blood pressure measurements before and 24 h after second dosing: No test item-related changes compared to saline and empty LNP control groups.<br>Hematology: Test-item related drop in lymphocyte counts at 2 days after administration. Levels normalized prior to day 9.<br>No noteworthy influence on clinical biochemistry parameters, or C3a and SC5b-9 complement factor plasma levels .
Tolerability study	Non-GLP	Cynomolgus monkeys	Monotherapy	10, 20 or 50 µg Alb-IL-2var	Day 1, 8	Tolerated. No test-item related systemic intolerance reactions. No deaths.<br>No influence on body weight, food consumption.<br>Hematology: Test-item related drop in lymphocyte counts with 20 and 50 µg Alb-IL-2var at 2 days after first administration. Levels normalized prior to second dosing. Slight changes for monocytes and basophils.<br>No test-item related changes in clinical biochemistry parameters.
Biomarker study	Non-GLP	Cynomolgus monkeys	Monotherapy	180 or 540 µg Alb-IL-2var	Day 1, 22	No systemic intolerance reactions.<br>Hematology: Marked test-item related drop in lymphocytes 1 day after administration to 50% of pre-dose values. 5 and 7 days after administration up to 4-fold increase with a consecutive drop to pre-dose levels around 14 – 21 days after administrations. Platelets, reticulocytes, monocytes, neutrophils, basophils with comparable pattern.<br>Flow cytometry: Marked increase in CD8^{+} T cells and NK cells 7 days after administration.<br>Anti-Alb-IL-2var protein ADA: No increase in IgG responses detectable. Weak, short-lived increase in IgM response compared to pre-dose sample in high dose cohort only.<br>Anti-PEG ADA: Detectable as early as 5 days after dosing. IgM responses increased dose-dependently and continuously dropped despite the second dose. IgG weakly detected in high dose cohort after second treatment only.

ADA = Anti-drug antibodies, Alb = Albumin, BALB/c = Bagg albino/c, C3a = Complement component 3a, GLP = Good laboratory practice, IgG = Immunoglobulin G, IgM = Immunoglobulin M, LNP = Lipid nanoparticle, LPX = Lipoplex, NA = Not applicable, NK = Natural killer, PD-1 = Programmed cell death protein 1, PEG = Polyethylene glycol, SC5b-9 = Soluble terminal complement complex C5b-9, Thy1.1 = Thymocyte antigen 1.1.
